# Tuning Structural Organization via Molecular Design and Hierarchical Assembly to Develop Supramolecular Thermoresponsive Hydrogels

**DOI:** 10.1021/acs.macromol.4c00567

**Published:** 2024-07-03

**Authors:** Dan Jing Wu, Martin G. T. A. Rutten, Jingyi Huang, Maaike J. G. Schotman, Johnick F. van Sprang, Bart M. Tiemeijer, Gijs M. ter Huurne, Sjors P. W. Wijnands, Mani Diba, Patricia Y. W. Dankers

**Affiliations:** † Laboratory for Cell and Tissue Engineering, Department of Biomedical Engineering, Eindhoven University of Technology, Eindhoven, PO Box 513 ,Eindhoven MB 5600, The Netherlands; ‡ Institute for Complex Molecular Systems, Eindhoven University of Technology, PO Box 513 ,Eindhoven MB 5600, The Netherlands; § Laboratory of Chemical Biology, Department of Biomedical Engineering, Eindhoven University of Technology, PO Box 513 ,Eindhoven MB 5600, The Netherlands; ∥ Laboratory of Macromolecular and Organic Chemistry, Department of Chemical Engineering and Chemistry, Eindhoven University of Technology, PO Box 513 ,Eindhoven MB 5600, The Netherlands; ⊥ Laboratory of Immunoengineering, Department of Biomedical Engineering, Eindhoven University of Technology, Eindhoven MB 5600, The Netherlands; # Department of Dentistry-Regenerative Biomaterials, Research Institute for Medical Innovation, Radboud University Medical Center, 6525EX ,Nijmegen 6500 HB, The Netherlands

## Abstract

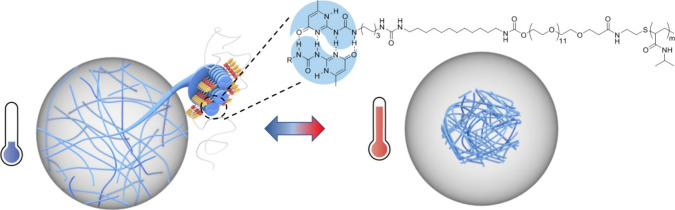

The cellular microenvironment is composed of a dynamic hierarchical fibrillar architecture providing a variety of physical and bioactive signals to the surrounding cells. This dynamicity, although common in biology, is a challenge to control in synthetic matrices. Here, responsive synthetic supramolecular monomers were designed that are able to assemble into hierarchical fibrous structures, combining supramolecular fiber formation via hydrogen bonding interactions, with a temperature-responsive hydrophobic collapse, resulting in cross-linking and hydrogel formation. Therefore, amphiphilic molecules were synthesized, composed of a hydrogen bonding ureido-pyrimidinone (UPy) unit, a hydrophobic alkyl spacer, and a hydrophilic oligo(ethylene glycol) tail. The temperature responsive behavior was introduced by functionalizing these supramolecular amphiphiles with a relatively short poly(*N*-isopropylacrylamide) (PNIPAM) chain (*M*_n_ ∼ 2.5 or 5.5 kg/mol). To precisely control the assembly of these monomers, the length of the alkyl spacer between the UPy moiety and PNIPAM was varied in length. A robust sol–gel transition, with the dodecyl UPy-PNIPAM molecule, was obtained, with a network elasticity enhancing over 2000 times upon heating above room temperature. The UPy-PNIPAM compounds with shorter alkyl spacers were already hydrogels at room temperature. The sol–gel transition of the dodecyl UPy-PNIPAM hydrogelator could be tuned by the incorporation of different UPy-functionalized monomers. Furthermore, we demonstrated the suitability of this system for microfluidic cell encapsulation through a convenient temperature sol–gel transition. Our results indicate that this novel thermoresponsive supramolecular system offers a modular platform to study and guide single-cell behavior.

## Introduction

The natural extracellular matrix (ECM) is largely composed of biomacromolecules that assemble into fibrillar structures, such as collagen, fibronectin, and elastin, forming hydrogel-like matrices.^1^ These matrices play a vital role in directing cellular behavior such as spreading, proliferation, differentiation, and migration.^2,3^ These cellular activities are orchestrated through a range of cues provided by the ECM, which play fundamental roles in the regulation of tissue development, maintenance/disease, and repair. The nature of these various cues arises through the fibrous nature of the ECM, impacting physical and mechanical properties as well as structural.^4^ Over the years, various strategies have been employed to mimic these properties into synthetic hydrogels for 3D cell culture and tissue engineering.^5,6^ Recently, there has been growing interest for supramolecular hydrogels,^7,8^ that can form fibrous structures^9^ via the self-assembly of small organic molecules,^10−12^ peptide amphiphiles,^13^ surfactant molecules,^14^ drug amphiphiles,^15^ or globular proteins^16^ into elongated, thin nanofibrils. These fibrillar systems can typically form a 3D hydrogel network, via branching, entanglement, physical associative interactions or a combination of these mechanisms, which can be tailored to control the mechanical properties of the hydrogels.^17^ The method of formulation and various external stimuli can be used to control gelation kinetics.^18−20^ A key advantage of supramolecular hydrogels is their dynamic and adaptable nature.^21−23^Owing to the reversibility of noncovalent bonds in these systems, matrix remodeling by cells can take place without the need for large pores or hydrogel degradation as required in conventional systems.^24−26^ Previously, we introduced a dynamic hydrogel system as synthetic ECM based on supramolecular building blocks containing ureido-pyrimidinone (UPy) motifs conjugated to an oligo(ethylene glycol) (OEG) or poly(ethylene glycol) (PEG) chain for water binding.^7^ These building blocks have been shown to assemble into long one-dimensional (1D) fibers in water, driven by UPy dimerization via quadruple hydrogen bonds as well as lateral stacking facilitated by flanking urea groups that create a hydrophobic pocket.^27−29^ Accordingly, at building block concentrations above the gelation threshold, bundling and entanglement of these supramolecular fibers results in the formation of a 3D hydrogel network.^11,30^ In previous studies, in order to tune the structural and kinetic properties of these UPy-based supramolecular assemblies, we demonstrated the use of molecular design parameters to develop functional monomers or mixing different monomer variants via a modular approach.^21,31,32^ Nonetheless, while the fibrous structure and dynamic nature of these materials greatly benefit their use as synthetic ECM, UPy-based hydrogel systems lack the capability to exhibit significant changes in their mechanical properties in response to temperature alterations that are compatible with living cells. This in large contrast to living tissue, where the ECM is stimuli-responsive and capable of adapting its mechanical properties in response to signals from their microenvironment.^33^

In this respect, an interesting synthetic material to incorporate dynamic properties is poly(*N*-isopropylacrylamide) (PNIPAM), which shows thermal responsive behavior in water, where it transitions form a hydrated to a dehydrated globule once heated above its lower critical solution temperature (LCST), i.e., ∼30 °C.^34^ This remarkable morphological transition makes it ideally suited for combinations with another stress-responsive system; as it can form a mechanical interlock to generate a stiffening effect. This was recently illustrated with semiflexible bis-urea-based-fibers, cross-linked with PNIPAM to yield temperature responsive and mechanically active hydrogel networks.^35^ Even more remarkable, without the need for covalent links, PNIPAM mixed with a stress-stiffening polyisocyanide (PIC) was able to generate a 50-fold increase of the original modulus when it collapsed, similar to the force exerted by myosin molecular motors.^36^

**Scheme 1:**
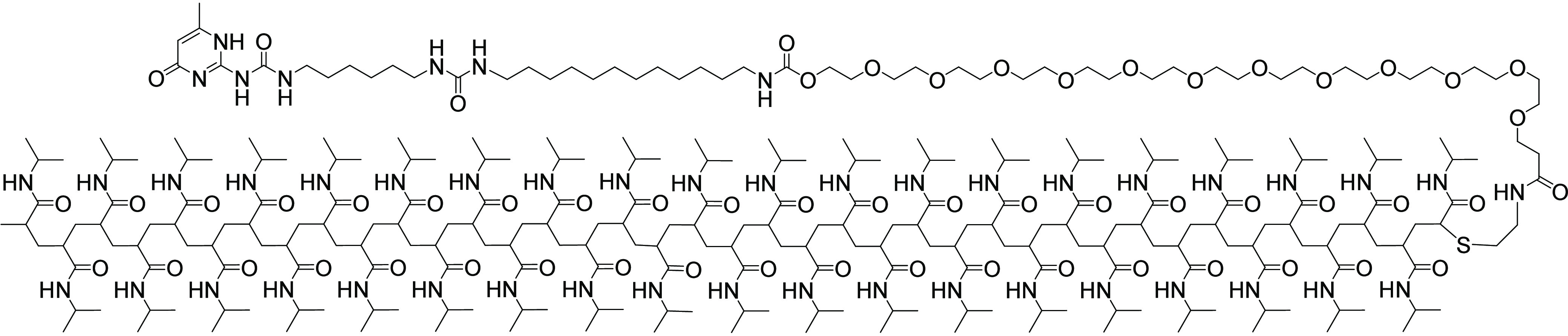
Chemical Structure of **UPy-C**_**12**_**–PNIPAM** (PNIPAM Being Polydisperse)

Here, we report a thermoresponsive UPy-based hydrogel system by designing supramolecular building blocks composed of PNIPAM (*M*_n_ = 2.5 kg/mol) functionalized with UPy moieties (Scheme 1). To study this system systematically, different variants of UPy-C_n_-PNIPAM (*n* = 8, 10, 12) monomers were designed, varying the alkyl spacer length, adjacent to the UPy (Scheme S1). Additionally, as a control, a UPy-PNIPAM monomer without an alkyl spacer was synthesized (**UPy-PNIPAM**). Also, a **UPy-C**_**12**_**–PNIPAM**_**5500**_ (*M*_n_ = 5.5 kg/mol) was designed to elucidate the influence of a very long PNIPAM chain on the supramolecular assembly of the monomers. The self-assembly behavior and thermoresponsiveness of this system at dilute conditions were elucidated using light scattering and various microscopy techniques. Moreover, viscoelastic properties and structure of assembled hydrogels were analyzed as a function of temperature by means of oscillatory rheology and small-angle X-ray scattering (SAXS). The modularity of this system enables a tunable sol–gel transition via the coassembly of different UPy-monomer variants. This modularity also allows facile introduction of bioactive moieties in the hydrogels by introduction of UPy-based monomers containing cell-adhesive ligands as supramolecular additives. Finally, as a proof-of-principle for application as synthetic ECM mimics, picoliter-sized fibrous hydrogel particles were fabricated via droplet microfluidics by leveraging the robust sol–gel transition in this system for encapsulation of single cells.

## Results and Discussion

### Design and Synthesis of UPy-C_n_-PNIPAM

To synthesize UPy-C_n_-PNIPAM with varying alkyl length (*n* = 8, 10, 12), UPy-C_*n*_-carboxylic acid precursor molecules (see Supporting Information) were reacted with the primary amine of amine-terminated PNIPAM (*M*_n_∼ 2.5 or 5.5 kg/mol). For this carboxamide formation, HATU and n-methylmorpholine were used as the coupling reagent and base, respectively, enabling the activation of the carboxylic acid for amine coupling (Scheme S4). Unreacted UPy-C_n_-carboxylic acid molecules were removed via extraction in 2-isopropyl ether. The excess amount of unreacted NH_2_–PNIPAM was removed by using a methyl-isocyanate resin. Accordingly, **UPy-C**_**8**_**–PNIPAM** was obtained with a yield of ∼98% (1.62 g). Using ^1^H NMR spectroscopy, the number-average molecular weight (*M*_n_) was determined at *M*_n_ = 2.9 kg/mol, meaning 14 PNIPAM repeating units on average. **UPy-C**_**10**_**-PNIPAM** was obtained with a yield of ∼97% (1.55 g), resulting in a *M*_n_ = 3.3 kg/mol based on 17 PNIPAM repeating units on average. **UPy-C**_**12**_**–PNIPAM** synthesis resulted in a yield of ∼97% (0.89 g) and *M*_n_ = 3.9 kg/mol based on 22 PNIPAM repeating units on average. To investigate the effect of PNIPAM chain length on the self-assembly of supramolecular fibers and the resulting hydrogels, **UPy-C**_**12**_**–PNIPAM**_**5500**_ was additionally synthesized with a longer PNIPAM chain length (*M*_n_ ∼ 5.5 kg/mol). Therefore, a similar synthesis protocol was used as described above, resulting in a yield of ∼46% (0.12 g) with *M*_n_ = 7.5 kg/mol based on 54 PNIPAM repeating units on average. As a control, **UPy-PNIPAM** molecules were synthesized by reacting the UPy-isocyanate precursor with the primary amine of amine-terminated PNIPAM (*M*_n_ ∼ 2.5 kg/mol) via base activation, after which a similar purification protocol was used as described above. This procedure resulted in a yield of ∼98% (1.15 g) with a *M*_n_ of 3.9 kg/mol based on 31 PNIPAM repeating units on average.

### Structural Characterization of Supramolecular Assemblies

**Figure 1:**
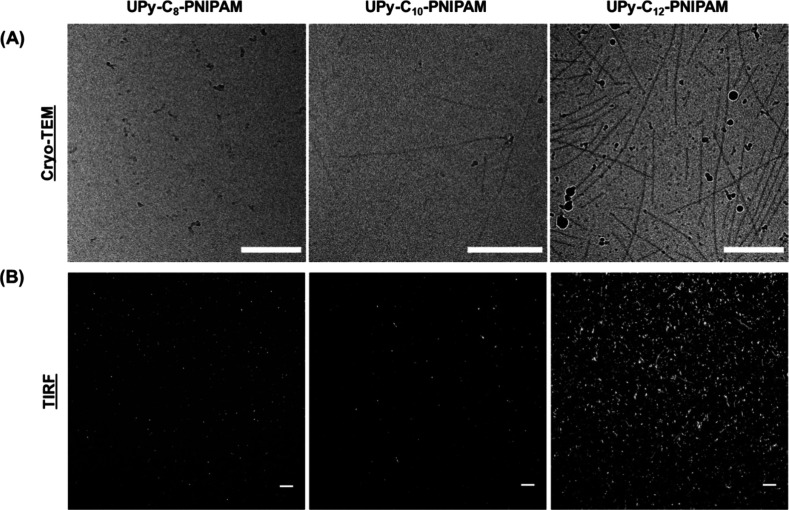
Microscopic characterization of supramolecular assemblies formed by UPy-C_n_-PNIPAM molecules. (A) Representative cryo-TEM images of globular structures formed by **UPy-C**_**8**_**–PNIPAM**, a mix of globular and fibrillar structures formed by **UPy-C**_**10**_**–PNIPAM**, and fibrillar structures formed by **UPy-C**_**12**_**–PNIPAM** molecules in aqueous solution (50 μM). The scale bars represent 200 nm. (B) Structural characterization of **UPy-C_*n*_–PNIPAM** in aqueous solutions (50 μM) using TIRF, demonstrating various structures for the **UPy-C_*n*_–PNIPAM** polymers. The increase in the alkyl-spacers resulted in an increase in the fiber length, indicating that UPy molecules can assemble into supramolecular fibers. The scale bars represent 5 μm.

The fibrous self-assembly in water of the various UPy-C_*n*_-PNIPAM molecules (Figure 1A) was studied by using cryogenic transmission electron microscopy (cryo-TEM). Cryo-TEM micrographs showed a characteristic nanofibrous organization for the UPy-modified monomers, in contrast to the pristine PNIPAM (Figure S2). These investigations revealed the formation of micrometers-long fibrillar structures with diameters of ∼5–7 nm. More specifically, assemblies composed of **UPy-C**_**8**_**–PNIPAM** displayed poorly defined globular aggregates with an average diameter of 6.3 ± 1.3 nm. In contrast, fibrous 1D nanostacking was observed for **UPy-C**_**10**_**-PNIPAM** and **UPy-C**_**12**_**–PNIPAM** assemblies. However, **UPy-C**_**10**_**-PNIPAM** also showed the formation of globular aggregates in addition to nanofibers with an average diameter of 5.6 ± 1.1 nm. Meanwhile, **UPy-C**_**12**_**–PNIPAM** displayed more defined and long nanofibers with an average diameter of 6.1 ± 1.1 nm. In contrast, pristine PNIPAM and **UPy-PNIPAM** did not show any structural organization in terms of fiber formation (Figure S2), likely due to the hydrophilic nature of PNIPAM at room temperature and the absence of a hydrophobic spacer in **UPy-PNIPAM**.

These results demonstrate that the length of the alkyl-spacer is a crucial factor to promote the self-assembly of the monomers in this supramolecular system as they shield the urea motives that induce stacking as well facilitate hydrophobic interactions between the molecules. Remarkably, shorter nanofibers with a higher polydispersity were observed for **UPy-C**_**12**_**–PNIPAM**_**5500**_ (average diameter = 4.9 ± 1.2 nm) when compared to **UPy-C**_**12**_**–PNIPAM**. This shows that the length of the covalently coupled PNIPAM chains affects the supramolecular fibrillar assembly of the UPy monomers and that the long PNIPAM chain might hamper tight packing and assembly of UPy monomers. To further investigate the effect of the PNIPAM functionalization on the self-assembly, **UPy-C**_**12**_**–OMe** molecules were analyzed. These molecules comprised the same self-assembling moieties but without PNIPAM functionalization and are known to form robust fibers, driven by an alkyl spacer that generates a hydrophobic pocket.^31^ These monomers assembled into micrometer-long well-defined nanofibers with an average diameter of 6.7 ± 1.1 nm (Figure S2). Overall, these results indicate the critical role of the hydrophobic alkyl spacer and the effect of PNIPAM functionalization on the assembly of supramolecular structures in this UPy-based system. Accordingly, delicate tuning of these two molecular features offers a new avenue to tune the assembly of supramolecular systems.

To gain a deeper understanding of the self-assembly of the designed supramolecular structures in aqueous media, different compositions of aggregates were also analyzed by using total internal reflection fluorescence microscopy (TIRF) (Figure 1B). To this end, the monomers were incubated with 5 mol % solvatochromic dye Nile Red (NR) overnight at a total concentration of 50 μM in an aqueous solution. Nile Red is an uncharged phenoxazone dye, which can be introduced as a fluorescent marker to indicate the polarity of the environment.^37^ As shown in Figure 1B, the results obtained in TIRF measurements were consistent with our observations in cryo-TEM experiments, revealing that **UPy-C**_**8**_**–PNIPAM** molecules assembled into globular aggregates, while **UPy-C**_**10**_**-PNIPAM** molecules formed both globular and fibrillar aggregates. For **UPy-C**_**12**_**–PNIPAM**, micrometer-long 1D fibrillar aggregates were obtained in a much higher amount than for the other molecules. This shows the crucial role of the C_12_ spacer in forming long nanofibers (cryo-TEM) that can bundle into larger fibrillar structures (TIRF). In contrast, the formation of supramolecular aggregates was not observed for **PNIPAM** and **UPy-PNIPAM** due to the hydrophilic nature of the polymers and their inferior capacity for stacking. In the absence of PNIPAM however, **UPy-C**_**12**_**–OMe** was able to form micrometers-long nanofibers and/or bundles, which corroborates the cryo-TEM results (Figures S2 and S3). Remarkably, the integration of the longer water-soluble PNIPAM chain in the **UPy-C**_**12**_**–PNIPAM**_**5500**_ molecules resulted in a disruption of stack formation, yielding significantly less and shorter fibers (Figure S3). Together, these results show the importance of the alkyl spacer in the assembly of supramolecular structures, showing that the longest alkyl-spacer (C_12_) favors the supramolecular interactions and allows the formation of long fibers.

**Figure 2:**
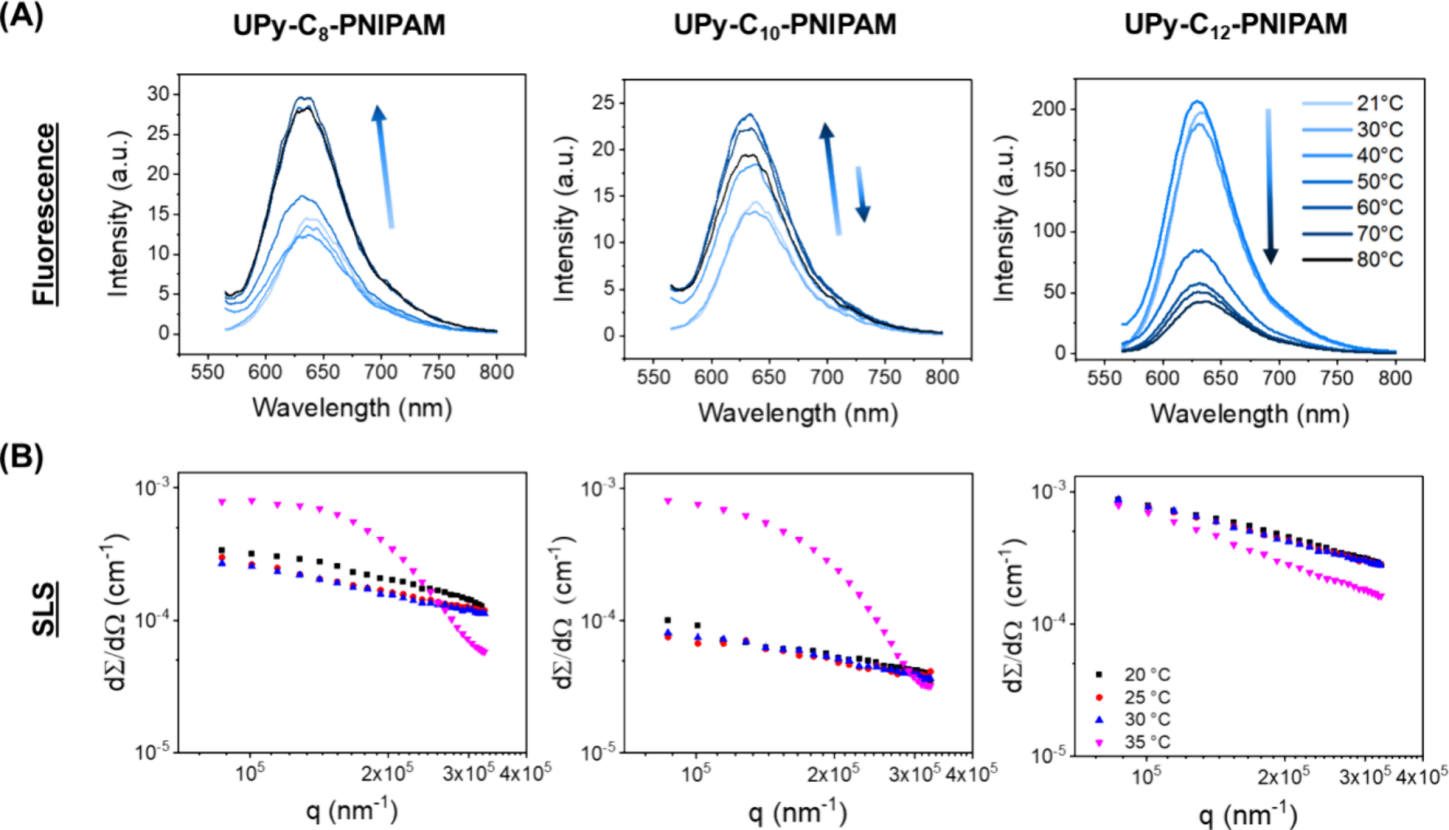
Spectroscopic characterization of supramolecular assemblies formed by UPy-C_n_-PNIPAM molecules at different temperatures. (A) Nile Red (NR) assay to investigate hydrophobicity in the polymers, showing a complex interplay between supramolecular fiber formation and the thermoresponsiveness of the PNIPAM (50 μM). Upon elevated temperatures, the PNIPAM transition caused an increase in the NR intensity for **UPy-C**_**8**_**–PINPAM** and **UPy-C**_**10**_**–PNIPAM**, indicating an increase in hydrophobicity. For **UPy-C**_**12**_**–PNIPAM**, the NR intensity decreased, indicating that the supramolecular interactions for fiber formation are dominating. Arrows indicate shift in NR intensity. (B) Structural characteristics of **UPy-C_*n*_–PNIPAM** assemblies were studied in aqueous solutions (50 μM) by using static light scattering (SLS). An increase of the alkyl spacer length resulted in preservation of the supramolecular fibers upon elevated temperatures, while short alkyl spacers showed a transition toward globular structures.

To investigate the relationship between the thermal response of PNIPAM and the supramolecular assembly of UPy-based building blocks, we further employed a NR fluorescence assay to investigate the hydrophobicity of the environment and to examine the change in hydrophobicity upon temperature variation (Figure 2A).^38^ In these experiments, a change in the emission intensity serves as an indicator for a change in the hydrophobic environment.^39^ For **UPy-C**_**8**_**–PNIPAM** and **UPy-C**_**10**_**–PNIPAM**, the maximum intensity increased at higher temperatures. This increase of NR fluorescence intensity upon temperature elevation indicates an increase in hydrophobicity due to the thermal response of PNIPAM. In contrast, **UPy-C**_**12**_**–PNIPAM** demonstrated a decrease in fluorescence intensity upon temperature elevation, which was also observed in **UPy-C**_**12**_**–PNIPAM**_**5500**_ and **UPy-C**_**12**_**–OMe** (Figure S4). This phenomenon shows that once the alkyl spacer is long enough, the supramolecular interactions that drive fiber assembly dominate over the temperature response of PNIPAM. That is, the NR response for PNIPAM functionalized monomers with a long alkyl spacer is similar to the UPy response without any PNIPAM functionalization. This was further supported by the pristine PNIPAM and **UPy-PNIPAM**, showing an increase of NR intensity upon temperature elevation, as now the thermoresponsive behavior of PNIPAM is dominating, as no supramolecular fibers can be formed (Figure S4). Furthermore, the different NR results for the various **UPy-C_*n*_–PNIPAM** molecules hint toward a different critical micelle concentration (CMC) for the various structures. The long C_12_ spacer likely results in a much lower CMC, as supramolecular fibers are formed more easily and dominate over the PNIPAM response. Collectively, the results of cryo-TEM, TIRF, and fluorescence spectroscopy indicate that the alkyl spacer included in our molecular design promotes the 1D fibrillar stacking of the monomers, with the C_12_ alkyl spacer displaying a particular tendency toward fiber assembly. Furthermore, NR fluorescence results supports these findings and demonstrate the interplay between the thermoresponsive behavior of PNIPAM and the supramolecular fiber formation, controlled via the length of this alkyl chain.

### Assessing Structural Transformation of Supramolecular Assemblies in Response to Temperature

Static light scattering (SLS) was used to derive possible changes in the structural configuration of supramolecular assemblies in response to temperature (Figure 2B). At 20 °C ≤ *T* ≤ 30 °C, the scattering profiles of the assemblies based on **UPy-C**_**8**_**–PNIPAM**, **UPy-C**_**10**_**-PNIPAM**, and **UPy-C**_**12**_**–PNIPAM**, all exhibited featureless slopes leveling off with a scattering intensity decay of roughly *q*^–1^, which is characteristic for fibrillar structures dispersed in an aqueous environment. However, at 35 °C, scattering curves corresponding to **UPy-C**_**8**_**–PNIPAM** and **UPy-C**_**10**_**-PNIPAM** displayed a steep decay at high *q* values, which indicated the presence of globular particles. This change of behavior at different temperatures can be attributed to the thermoresponsive properties of the PNIPAM. Interestingly, this phenomenon did not manifest in the **UPy-C**_**12**_**–PNIPAM**, where the scattering curves exhibits only a small decrease at 35 °C, indicating that the fiber-like structure is preserved at the various temperatures, with possibly a marginal effect from PNIPAM collapse at 35 °C. This result was consistent with the structural results obtained by TIRF and fluorescence spectroscopy measurements. Taken together, our observations indicate that **UPy-C**_**8**_**–PNIPAM** and **UPy-C**_**10**_**–PNIPAM** form short nanofibers compared to **UPy-C**_**12**_**–PNIPAM**, at temperatures below the transition of PNIPAM. Once heated, the collapse of PNIPAM causes the structures of **UPy-C**_**8**_**–PNIPAM** and **UPy-C**_**10**_**–PNIPAM** to collapse into globular aggerates. In contrast, for **UPy-C**_**12**_**–PNIPAM**, the balance between the hydrophobic alkyl spacer and the length of the water-soluble PNIPAM results in robust nanofiber stacks with thermosensitive ends, which preserve the fibrillar architecture at higher temperature.

### Viscoelastic and Thermal Behavior of Hydrogels

**Figure 3:**
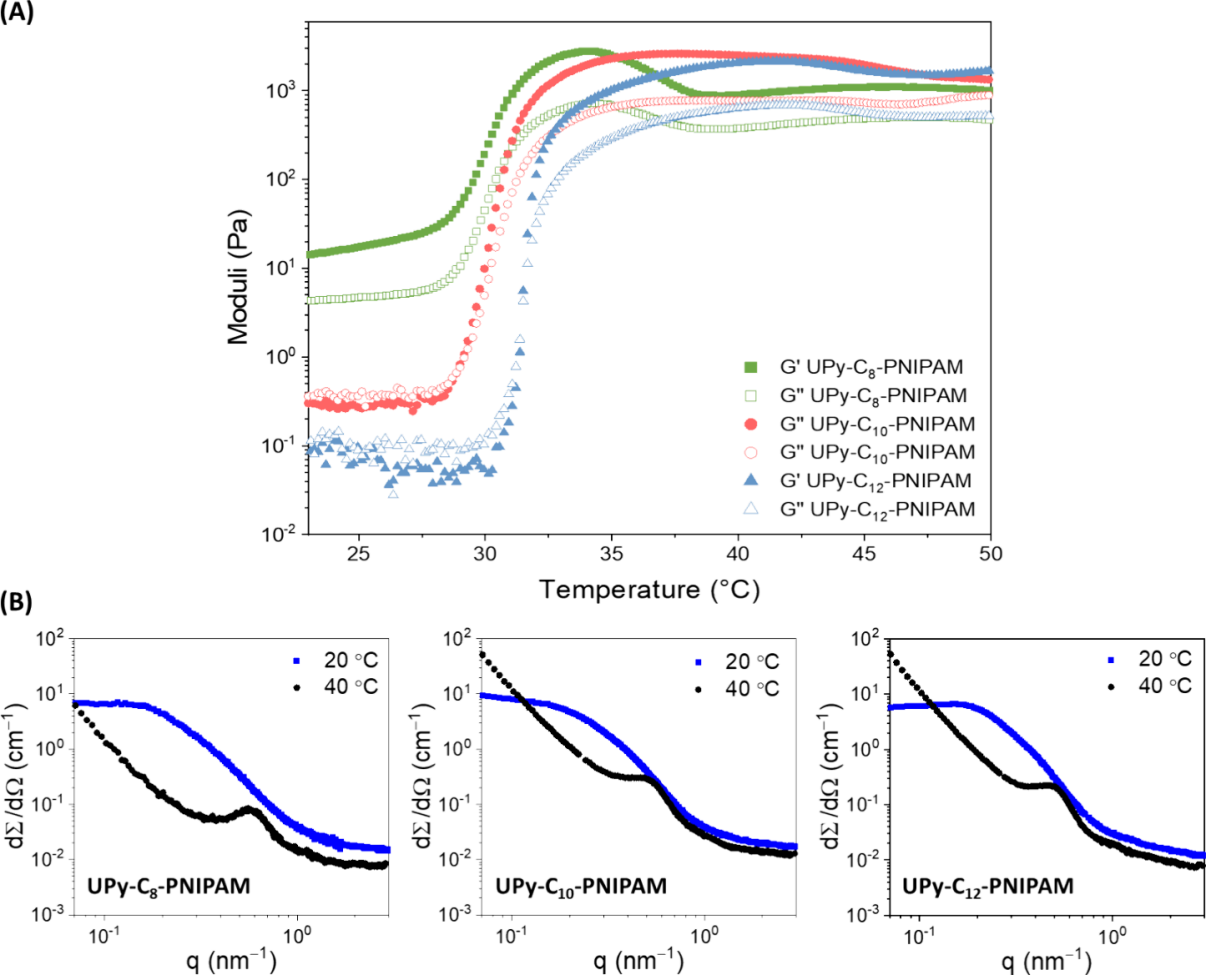
Thermoresponsive mechanical and structural behavior of **UPy-C_*n*_–PNIPAM** in an aqueous environment. (A) Sharp mechanical transition, as a result of temperature change was observed for the **UPy-C_*n*_–PNIPAM** polymers using rheology. The incorporation of a longer alkyl spacer demonstrates a softening in the material and can even induce a liquid-like (*G*″ > *G*′) response at room temperature. In contrast, a sharper mechanical transformation is observed for a longer alkyl spacer during the temperature increase. All samples contained 10 wt % solid content. (B) Small-angle X-ray scattering (SAXS) profile of 10 wt % compositions at low (20 °C) and high (40 °C) temperature. Comparison of the scattering intensities of the 10 wt % **UPy-C_*n*_–PNIPAM** compositions at 20 and 40 °C to probe the structural organization and the mechanism of temperature transition.

We next studied the viscoelastic properties of this supramolecular system at various temperatures by increasing the concentration of monomers from a dilute condition, used in the above-described experiments (1.8 × 10^–5^ wt %), to a higher concentration (10 wt %), where UPy fibers are able to entangle and form a hydrogel.^7,23^ At room temperature, this resulted in a noteworthy difference in materials’ behavior for the various UPy-C_n_-PNIPAM molecules, most likely related to the size of self-assembled fibers. Whereas **UPy-C**_**8**_**–PNIPAM** and **UPy-C**_**10**_**–PNIPAM** were able to form stable (weak) gels, **UPy-C**_**12**_**–PNIPAM** failed in an inverted-vial test (Figure S5). Subsequently, to gain further insight into the viscoelastic properties of the samples composed of **UPy-C_*n*_–PNIPAM** molecules, oscillatory rheological measurements were conducted with a gradual temperature increase from 20 to 50 °C. Rheological characterizations of these compositions revealed that while both **UPy-C**_**12**_**–PNIPAM** and **UPy-C**_**10**_**–PNIPAM** show liquid-like behavior (*G*′′ > *G*′) at room temperature, **UPy-C**_**8**_**–PNIPAM** formed a hydrogel exhibiting solid-like response (*G*′ > *G*′′) (Figure 3A). Upon increasing temperatures, all supramolecular compositions displayed a sharp increase in their viscoelastic moduli near the transition temperature of PNIPAM (∼30 °C), which was most remarkable for the **UPy-C**_**12**_**–PNIPAM**, as this composition was able to rapidly undergo a mechanically transformation from liquid (*G*′ < *G*′′) to gel (*G*′ > *G*′′) (Figure 3A). Interestingly, the sol–gel transition for this composition occurred almost instantly, where *G*′ increased almost over 200 times within one °C (in 1 min) and even over 2000 times within 10 °C (in 10 min), demonstrating the strength of the thermoresponsive behavior in this system. Although less pronounced than for **UPy-C**_**12**_**–PNIPAM**, **UPy-C**_**10**_**-PNIPAM**, and **UPy-C**_**8**_**–PNIPAM** also exhibited a sharp and well-defined thermoresponsive behavior as their *G*′ increased over 100 times within 10 °C.

The differences in mechanical and thermal behavior in these hydrogels can be attributed to the structural differences in the assembly of the supramolecular fibers directed by the alkyl spacer incorporated in the molecular designs. For **UPy-C**_**8**_**–PNIPAM** and **UPy-C**_**10**_**-PNIPAM**, small supramolecular aggregates are present resulting in superior viscoelastic moduli at room temperature compared to **UPy-C**_**12**_**–PNIPAM**. Hence, the latter exhibits long flexible nanofibers with hydrophilic (below the LCST) PNIPAM chains at the periphery. This design results in a flexible and hydrated structure that is proposed to freely diffuse in solution at room temperature. However, this transforms rapidly into a solid-like material (*G*′ > *G*″) upon an increase of temperature. This remarkable temperature responsiveness is due to the organization of the PNIPAM side chains into long fibers, yielding an ordered structure of PNIPAM chains. Upon heating, these PNIPAM side chains can interact with one another and form cross-links between the UPy fibers, rendering a hydrogel network.

In contrast to the UPy-C_n_-PNIPAM compositions, the 10 wt % pristine PNIPAM composition does not form a hydrogel at room temperature and does also not display a sol–gel transition upon temperature increase (Figure S6A). This phenomenon arises from the inability of pristine PNIPAM to form a continuous 3D network (*G*′ < *G*′′) upon heating, where instead phase separation occurs and consequent precipitation of the polymer phase. A similar phenomenon was observed for **UPy-PNIPAM** without an alkyl spacer, demonstrating the lack of a preorganization in terms of a fibrous structure, which is in agreement with the previous TIRF and fluorescent measurement results (Figure S6A). Moreover, a 10 wt % **UPy-C**_**12**_**–OMe** composition, without PNIPAM functionalization, formed a solid-like hydrogel at room temperature, with only a limited increase in viscoelastic moduli upon temperature increase when compared to UPy-C_n_-PNIPAM compositions. It is noteworthy that the **UPy-C**_**12**_**–OMe** composition exhibited a higher elastic response compared to the **UPy-C_*n*_–PNIPAM** compositions at different temperatures, which can be attributed to the absence of PNIPAM in **UPy-C**_**12**_**–OMe** samples, resulting in a higher amount of UPy-moieties available for interfiber cross-links.

Finally, to investigate the effect of PNIPAM chain length on viscoelastic behavior of the hydrogels, **UPy-C**_**12**_**–PNIPAM**_**5500**_ was also studied by using rheology (Figure S6B). These experiments revealed a *G*′ value for this material more than 10 times lower than that of **UPy-C**_**12**_**–PNIPAM** once heated just above the LCST of PNIPAM. Furthermore, when the temperature is raised further, *G*′ drops significantly until the point where **UPy-C**_**12**_**–PNIPAM**_**5500**_ undergoes a gel-to-sol transition (∼40 °C). These results indicate the importance of PNIPAM chain length in this design, and the lower mechanical stability of **UPy-C**_**12**_**–PNIPAM**_**5500**_ is likely related to the lower availability of UPy moieties that can form cross-links, due to the presence of a longer PNIPAM chain.

### Structural Characterization of Hydrogels

To obtain further insight into the nanostructural organization and the temperature correlated transition of 10 wt % **UPy-C_*n*_–PNIPAM** compositions, small-angle X-ray scattering (SAXS) measurements were carried out at 20 and 40 °C. At 20 °C, the scattering profiles for all the **UPy-C_*n*_–PNIPAM** compositions showed a featureless decay with steep slopes (Figure 3B), indicating a fibrous configuration with long stretched structures that supports the previous structural and microscopy results. Moreover, a similar profile was observed for **UPy-OMe** controls at 20 °C, whereas **UPy-PNIPAM** and pristine PNIPAM compositions displayed signals typical for elongated systems (Figure S7).^40^ Upon heating beyond the transition temperature of PNIPAM, at 40 °C, a scattering intensity peak appeared for all **UPy-C_*n*_–PNIPAM** compositions, as heterogeneities in the network architecture become more pronounced. This change is attributed to a transformation in the hydrogel network architecture, where PNIPAM chains collapse and interact upon heating. The SAXS measurement on **UPy-C**_**8**_**–PNIPAM**, **UPy-C**_**10**_**–PNIPAM**, and **UPy-C**_**12**_**–PNIPAM** hydrogels showed a maximum reflection peak at q* = 0.59 nm^–1^_,_ 0.52 nm^–1^_,_ and 0.50 nm^–1^ with a domain spacing (d*) of 10.6, 12.1, and 12.6 nm, respectively. These characteristics are most likely attributed to the PNIPAM chains, which collapse into coils at high temperature and segregate from the UPy fibers. Further insight in these UPy fibers was obtained from the SAXS measurement on **UPy-C**_**12**_**–OMe** (Figure S7), which demonstrated a primary reflection peak at q* = 0.39–0.46 nm^–1^ with a higher order reflection at q* = 2.93 nm^–1^. These reflections correspond to domain spacing (d*) of 16.1–13.6 nm, which is possibly caused by the PEG chains that segregate from the UPy fibers. Collectively, these findings establish the structural organization of the **UPy-C_*n*_–PNIPAM** hydrogels, which show characteristics of the PNIPAM collapse between the UPy fibers or the interfiber distance.

Altogether, these results are in line with the previous analysis of polymer assembly in dilute conditions (Figures 1 and 2). Ultimately, a long enough hydrophobic spacer adjacent to a UPy moiety is required to obtain long fibers, which preorganize the PNIPAM side chains, thereby yielding a strong and sharp mechanical response upon elevated temperatures via mechanical interlocking of PNIPAM. The importance of this preorganization was further proven by formulating 10 wt % samples composed of **UPy-C**_**12**_**–OMe** and pristine PNIPAM (*M*_n_ ∼ 2.5 kDa), without a covalent link between the two molecules. As shown in Figure S6B, these samples exhibited a much weaker increase in viscoelastic moduli upon heating, highlighting the importance of this covalent linkage between the PNIPAM and UPy moiety and the preorganization. Finally, we observed that longer PNIPAM chain disrupts the UPy-nanofiber preorganization, which results in the formation of weaker hydrogel networks, showing a delicate balance between hydrophobic and hydrophilic moieties, which control the formation and nature of the formed networks.

### Concentration- and Time-Dependent Thermoresponsive Behavior of Hydrogels

To gain deeper insight into the importance of organizing the PNIPAM chains into fibrillar structures, a range of concentrations and preassembly times were studied. Therefore, **UPy-C**_**12**_**–PNIPAM** was used, as it previously showed the sharpest thermo-mechanical response. Rheological measurements using different concentrations of **UPy-C**_**12**_**–PNIPAM** demonstrated the tunability of hydrogels’ elasticity by varying the **UPy-C**_**12**_**–PNIPAM** concentration (0.42 wt %, 0.84 wt %, 1.27 wt %, 1.68 wt %, 3.32 wt %) (Figure S8B). At *T* > 35 °C, hydrogels composed of higher monomer concentrations displayed higher *G*′ values, which is a common behavior for hydrogel systems.^7^ More interestingly, viscoelastic behavior of the samples varied as a function of the preassembly time of monomers prior to the rheological measurements (Figure S8C). To clarify, **UPy-C**_**12**_**–PNIPAM** samples were prepared by dissolving the solid molecules at high temperatures and pH, yielding small aggregates. Subsequent neutralization allows reformation of hydrogen bonds between the UPy moieties and subsequent formation of fibrillar structures. Clearly, a longer assembly time allows formation of longer fibers, and thus a more ordered network of the PNIPAM chains, which leads to a stronger increase in the elasticity of the networks once heated above the LCST of PNIPAM.

Finally, we investigated the reversibility of the sol–gel transition behavior of **UPy-C**_**12**_**–PNIPAM** compositions (1.25 wt %) over multiple heating–cooling cycles. As shown in Figure S8D, these fibrous structures exhibited reversible elasticity over multiple cycles of low (10 °C) and high (40 °C) temperatures. Only above the LCST of PNIPAM a strong elastic like hydrogel network is formed while below this temperature, the network becomes softer. Interestingly, our results demonstrated an increase of *G*′ upon applying the first heating–cooling cycle, but particularly in the second 40 °C phase, in which the *G*′ value was ∼3 times higher than the value in the first 40 °C phase. Indicating that upon initial collapse and cooling, part of the structure is preserved, which can enhance the moduli of the network upon repeated heated and cooling cycles.

### Modular Tuning of Thermoresponsive Behavior of Hydrogels

**Figure 4:**
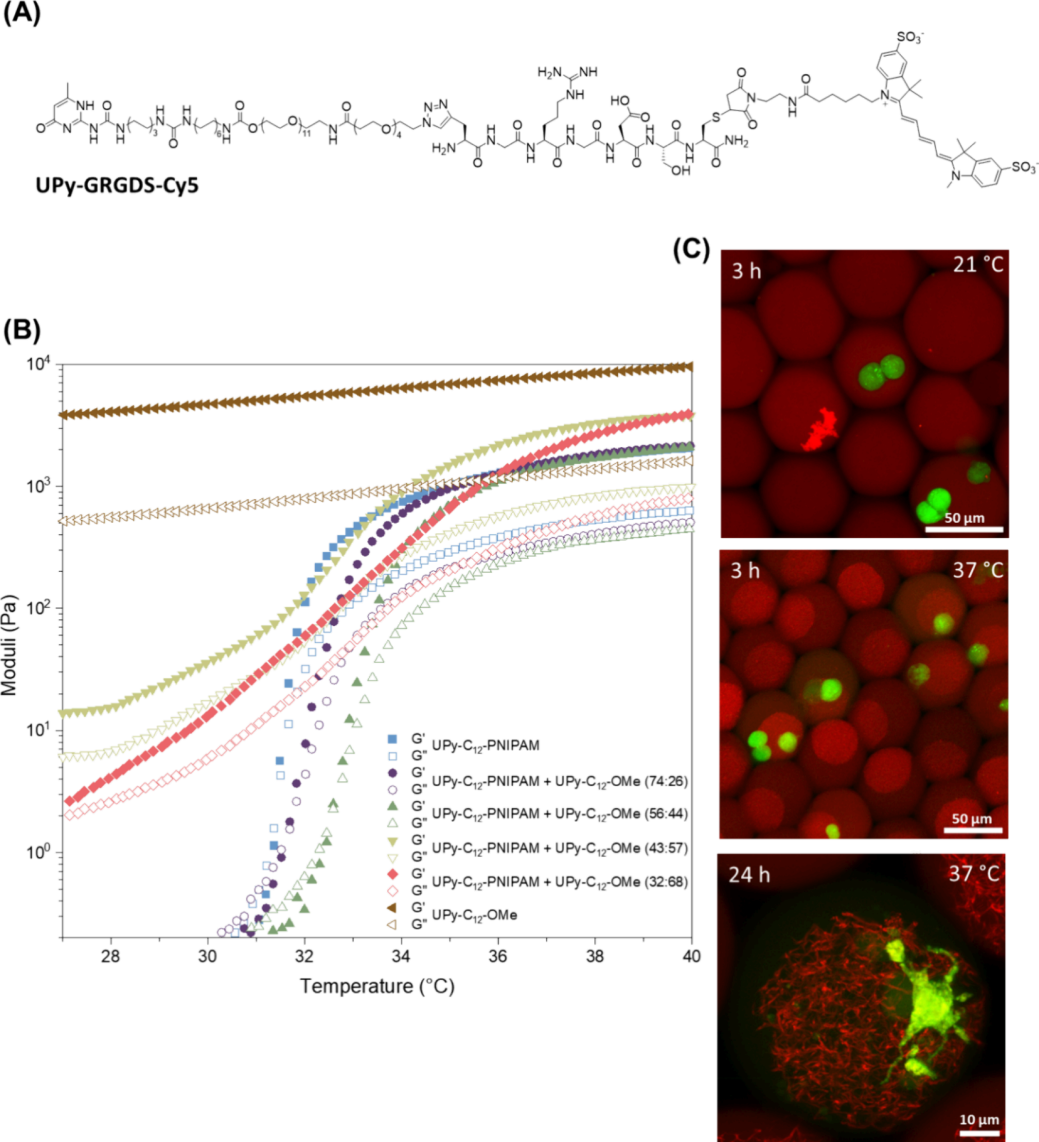
Modular tailoring of the biological or viscoelasticity and thermal response of fibrous hydrogels through coassembly of UPy-C_12_–PNIPAM with UPy-OMe or UPy-GRGDS-Cy5 additives. (A) Chemical structure of UPy-GRGDS-Cy5. (B) Rheological measurements of 10 wt % hydrogels of **UPy-C**_**12**_**–PNIPAM**, **UPy-OMe**, and their combinations (molar ratios between brackets). Increasing the **UPy-C**_**12**_**–OMe** in the molar ratio increased the storage modulus of the resulting hydrogels. In addition, a shift in the transition response was induced by increasing **UPy-C**_**12**_**–OMe** content. When the hybrid system contained more than 50 mol % **UPy-C**_**12**_**–OMe**, a gel was formed at room temperature (*G*′ > *G*′′). **C. UPy-GRGDS-Cy5** was coassembled with **UPy-C**_**12**_**–PNIPAM** in a molar ratio of 1:50. Human induced pluripotent stem cells (hiPSCs) were encapsulated in hydrogel droplets of 1.25 wt %, via a microfluidic system. At 21 °C, monodisperse droplets were formed with a homogeneous distribution of **UPy-GRGDS-Cy5** throughout the droplet. In contrast, hydrogel formation was evident at 37 °C as indicated by colocalization of the **UPy-GRGDS-Cy5** additive. Fibrous features in the hydrogel were maintained after 24 h of incubation. The incorporation of **UPy-GRGDS-Cy5** into the fibrillar hydrogel resulted in cell spreading.

We next explored the capacity of this supramolecular system to exhibit tunable thermoresponsive behavior via the modular combination of different self-assembling monomers. To this end, we explored hydrogel formation (10 wt %) based on coassembly of **UPy-C**_**12**_**–OMe** and **UPy-C**_**12**_**–PNIPAM** monomers at varying ratios (Figure 4). Interestingly, our results revealed that by increasing the **UPy-C**_**12**_**–OMe** content relative **to UPy-C**_**12**_**–PNIPAM**, the sol–gel temperature of the system shifts toward higher temperatures (Figure 4B). This shift was evident in hydrogels with up to 44 mol % **UPy-C**_**12**_**–OMe** content (i.e., 56/44 **UPy-C12-PNIPAM**/**UPy-C**_**12**_**–OMe** molar ratio), above which the samples were already in a gel state (*G*′ > *G*″) at room temperature and exhibited an inferior thermal response. In addition, hydrogels with a higher **UPy-C**_**12**_**–OMe** content exhibited a higher elastic modulus at a high temperature (e.g., 37 °C). Overall showing that by delicate tuning of the composition, a certain desired balance between a strong thermal response and high elastic modulus can be created (Figure 4B).

Collectively, our results highlight the capacity of this UPy-based system for enhanced control over hydrogel formation and their sol–gel transition by means of (i) the molecular design as well as (ii) modular coassembly of various building blocks. These findings shed light on the relationship between the hydrogel structure and the mechanism of gelation in supramolecular fibrillar hydrogels, offering new avenues for controlling the viscoelasticity and the sol–gel transition.

### Cell Adhesion in Picoliter-Sized Fibrillar Hydrogel Beads

Natural ECM employs combinations of different chemical building blocks to tune the local extracellular environment.^41^ Droplet microfluidic approaches have recently demonstrated the potential to serve as powerful platforms in studying and decoding biological models at single-cell resolution.^42−45^ These systems typically rely on the use of hydrogels as ECM mimics for the encapsulation of cells. Therefore, we investigated the capacity of the UPy-based hydrogel to deliver a tunable ECM environment for microfluidic encapsulation and culture of human induced pluripotent stem cells (hiPSC). To this end, a droplet-based microfluidic approach was used to enable the production of highly controlled picolitre-sized hydrogel beads (∼70 pL) with reproducible structure and composition.^46^ The specific setup and settings were chosen to achieve the highest possible single cell encapsulation (∼20%).^46^

In order to make the hydrogel suitable for cell adhesion, GRGDS moieties can be incorporated. Previous studies already showed the effectiveness of incorporating these bioactive ligands into UPy hydrogels to control cellular spreading in bulk.^7,47^ Therefore, in our system, UPy-functionalized Gly-Arg-Gly-Asp-Ser (GRGDS)^48^ cell-adhesive ligands were incorporated into the **UPy-C**_**12**_**–PNIPAM** hydrogel via a modular approach. Moreover, these UPy-GRGDS molecules were labeled with a sulfocyanine5 (Cy5) dye (Figure 4A) to visualize the bioactive functionalization of the nanofibers. For the preparation of hydrogel precursor solutions, the **UPy-GRGDS-Cy5** additive (Figure 4C) was mixed with **UPy-C**_**12**_**–PNIPAM** in a molar ratio of 1:50. At 21 °C, monodisperse droplets were produced with a homogeneous distribution of in total 1.25 wt % UPy-based molecules. As expected, at 37 °C, a visible collapse in the polymer fibrous structure was observed, indicating hydrogel formation, which is in accordance with the rheological measurements. Furthermore, the hydrogel bead maintained the fibrous structure for at least 24 h of incubation. Importantly, after 24h, morphological changes of iPSCs were observed, indicating cell–matrix interactions. This spreading behavior confirmed the suitability of our hydrogel to study its effects on cell adhesion, spreading, and interaction at a single-cell level (Figure 4C, Video S1). This spreading behavior not only confirmed the availability of stable RGD ligands for cell adhesion in the hydrogel network but also indicated the adaptability of the supramolecular fibers, allowing local remodeling by the cells. Altogether, these results demonstrate that the tunable self-assembly of the fibrous **UPy-C**_**12**_**–PNIPAM** hydrogel exhibits great potential as a biomaterial template, allowing the investigation of cellular responses in a dynamic and responsive ECM environment at single-cell resolution.

## Conclusions

In this study, we showed the supramolecular design of fibrous hydrogels with robust thermoresponsive behavior by tailoring the molecular structure of building blocks containing UPy motifs and PNIPAM. Our results reveal that the increase in the length of the hydrophobic alkyl spacer adjacent to the UPy moiety directs the supramolecular assembly toward the formation of nanofibrillar structures. Our experimental studies of **UPy-C_*n*_–PNIPAM** molecules in dilute conditions underscore the importance of achieving a delicate balance between the hydrophobic UPy-based moieties and the length of water-soluble PNIPAM for effective fibrillar assembly based on these building blocks. Moreover, rheological measurements show that the coupling of the UPy moiety and PNIPAM is essential for achieving a robust thermal response in this system. Finally, our proof-of-concept cell experiment demonstrates the strong potential of this hydrogel system for the microfluidic encapsulation of cells.

## Supporting Information Available

Materials and methods; synthetic route for the various UPy’s; cell culture; microfluidic production; additional Cryo-TEM images; additional TIRF images; additional Nile red experiments; inverted vial test; additional rheology experiments; additional SAXS measurements (PDF)
Additional z-stack image, which rotates around hydrogel beads with encapsulated cell (AVI)

## Supplementary Material

ma4c00567_si_001.pdf

ma4c00567_si_002.avi

## ABBREVIATIONS

ureido-pyrimidinone: UPy
poly(*N*-isopropylacrylamide): PNIPAM
extracellular matrix: ECM
oligo(ethylene glycol): OEG
poly(ethylene glycol): PEG
lower critical solution temperature: LCST
polyisocyanide: PIC
small-angle X-ray scattering: SAXS
number-average molecular weight: Mn
cryogenic transmission electron microscopy: Cryo-TEM
total internal reflection fluorescence microscopy: TIRF
Nile Red: NR
critical micelle concentration: CMC
Static light scattering: SLS
human induced pluripotent stem cells: hiPSC
sulfo-cyanine5: Cy5

## References

[ref1] Theocharis A. D.; Skandalis S. S.; Gialeli C.; Karamanos N. K. Extracellular matrix structure. Adv. Drug Delivery Rev. 2016, 97, 4–27. 10.1016/j.addr.2015.11.001. 26562801

[ref2] Cushing M. C.; Anseth K. S. Hydrogel cell cultures. Science 2007, 316, 1133–1134. 10.1126/science.1140171. 17525324

[ref3] Kopeček J. Swell gels. Nature 2002, 417, 389–391. 10.1038/417388a. 12024197

[ref4] Doyle A. D.; Wang F. W.; Matsumoto K.; Yamada K. M. One-dimensional topography underlies three-dimensional fibrillar cell migration. J. Cell Biol. 2009, 184, 481–490. 10.1083/jcb.200810041. 19221195 PMC2654121

[ref5] Prince E.; Kumacheva E. Design and applications of man-made biomimetic fibrillar hydrogels. Nat. Rev. Mater. 2019, 4, 99–115. 10.1038/s41578-018-0077-9.

[ref6] Blache U.; et al. Engineered hydrogels for mechanobiology. Nat. Rev. Methods Primers 2022, 2, 98 10.1038/s43586-022-00179-7. 37461429 PMC7614763

[ref7] Diba M.; et al. Engineering the Dynamics of Cell Adhesion Cues in Supramolecular Hydrogels for Facile Control over Cell Encapsulation and Behavior. Adv. Mater. 2021, 33, 2008111 10.1002/adma.202008111. PMC11468203 34337776

[ref8] Webber M. J.; Appel E. A.; Meijer E. W.; Langer R. Supramolecular biomaterials. Nat. Mater. 2016, 15, 13–26. 10.1038/nmat4474. 26681596

[ref9] Weiss R. G.; Terech P. Molecular Gels. Molecular Gels (Springer, 2006). doi:10.1007/1-4020-3689-2.

[ref10] Estroff L. A.; Hamilton A. D. Water gelation by small organic molecules. Chem. Rev. 2004, 104, 1201–1217. 10.1021/cr0302049. 15008620

[ref11] Dankers P. Y. W.; et al. Hierarchical formation of supramolecular transient networks in water: a modular injectable delivery system. Adv. Mater. 2012, 24, 2703–2709. 10.1002/adma.201104072. 22528786

[ref12] Chebotareva N.; Bomans P. H. H.; Frederik P. M.; Sommerdijk N. A. J. M.; Sijbesma R. P. Morphological control and molecular recognition by bis-urea hydrogen bonding in micelles of amphiphilic tri-block copolymers. Chem. Commun. 2005, 4967–4969. 10.1039/b507171b. 16205816

[ref13] Webber M. J.; Berns E. J.; Stupp S. I. Supramolecular nanofibers of peptide amphiphiles for medicine. Isr. J. Chem. 2013, 53, 530–554. 10.1002/ijch.201300046. 24532851 PMC3922220

[ref14] Raghavan S. R. Distinct character of surfactant gels: A smooth progression from micelles to fibrillar networks. Langmuir 2009, 25, 8382–8385. 10.1021/la901513w. 19537741

[ref15] Lock L. L.; et al. Self-assembly of natural and synthetic drug amphiphiles into discrete supramolecular nanostructures. Faraday Discuss. 2013, 166, 285–301. 10.1039/c3fd00099k. 24611283 PMC3955011

[ref16] Xu J.; Palmer A.; Wirtz D. Rheology and microrheology of semiflexible polymer solutions: Actin filament networks. Macromolecules 1998, 31, 6486–6492. 10.1021/ma9717754.

[ref17] Raghavan S. R.; Douglas J. F. The conundrum of gel formation by molecular nanofibers, wormlike micelles, and filamentous proteins: Gelation without cross-links?. Soft Matter 2012, 8, 8539–8546. 10.1039/c2sm25107h.

[ref18] Niece K. L.; et al. Modification of gelation kinetics in bioactive peptide amphiphiles. Biomaterials 2008, 29, 4501–4509. 10.1016/j.biomaterials.2008.07.049. 18774605 PMC2584653

[ref19] Otsuka T.; Maeda T.; Hotta A. Effects of Salt Concentrations of the Aqueous Peptide-Amphiphile Solutions on the Sol–Gel Transitions, the Gelation Speed, and the Gel Characteristics. J. Phys. Chem. B 2014, 118, 11537–11545. 10.1021/jp5031569. 25196562

[ref20] Ozbas B.; Kretsinger J.; Rajagopal K.; Schneider J. P.; Pochan D. J. Salt-Triggered Peptide Folding and Consequent Self-Assembly into Hydrogels with Tunable Modulus. Macromolecules 2004, 37, 7331–7337. 10.1021/ma0491762.

[ref21] Dankers P. Y. W.; Meijer E. W. Supramolecular biomaterials. a modular approach towards tissue engineering. Bull. Chem. Soc. Jpn. 2007, 80, 2047–2073. 10.1246/bcsj.80.2047.

[ref22] Kieltyka R. E.; et al. Mesoscale modulation of supramolecular ureidopyrimidinone-based poly(ethylene glycol) transient networks in water. J. Am. Chem. Soc. 2013, 135, 11159–11164. 10.1021/ja403745w. 23829684

[ref23] Pape A. C. H.; et al. Mesoscale characterization of supramolecular transient networks using SAXS and rheology. Int. J. Mol. Sci. 2014, 15, 1096–1111. 10.3390/ijms15011096. 24441567 PMC3907858

[ref24] Chaudhuri O.; et al. Substrate stress relaxation regulates cell spreading. Nat. Commun. 2015, 6, 6365 10.1038/ncomms7365. PMC4518451 25695512

[ref25] Wang H.; Heilshorn S. C. Adaptable Hydrogel Networks with Reversible Linkages for Tissue Engineering. Adv. Mater. 2015, 27, 3717–3736. 10.1002/adma.201501558. 25989348 PMC4528979

[ref26] Diba M.; et al. Self-Healing Biomaterials: From Molecular Concepts to Clinical Applications. Adv. Mater. Interfaces 2018, 5, 1800118 10.1002/admi.201800118.

[ref27] Kautz H.; Van Beek D. J. M.; Sijbesma R. P.; Meijer E. W. Cooperative end-to-end and lateral hydrogen-bonding motifs in supramolecular thermoplastic elastomers. Macromolecules 2006, 39, 4265–4267. 10.1021/ma060706z.

[ref28] Appel W. P. J.; Portale G.; Wisse E.; Dankers P. Y. W.; Meijer E. W. Aggregation of ureido-pyrimidinone supramolecular thermoplastic elastomers into nanofibers: A kinetic analysis. Macromolecules 2011, 44, 6776–6784. 10.1021/ma201303s.

[ref29] van Beek D. J. M.; Spiering A. J. H.; Peters G. W. M.; Nijenhuis K.; Sijbesma R. P. Unidirectional Dimerization and Stacking of Ureidopyrimidinone End Groups in Polycaprolactone Supramolecular Polymers. Macromolecules 2007, 40, 8464–8475. 10.1021/ma0712394.

[ref30] Bastings M. M. C.; et al. A Fast pH-Switchable and Self-Healing Supramolecular Hydrogel Carrier for Guided, Local Catheter Injection in the Infarcted Myocardium. Adv. Healthc. Mater. 2014, 3, 70–78. 10.1002/adhm.201300076. 23788397

[ref31] Hendrikse S. I. S. S.; et al. Controlling and tuning the dynamic nature of supramolecular polymers in aqueous solutions. Chem. Commun. 2017, 53, 2279–2282. 10.1039/C6CC10046E. 28154855

[ref32] Rutten M. G. T. A.; Rijns L.; Dankers P. Y. W. Controlled, supramolecular polymer formulation to engineer hydrogels with tunable mechanical and dynamic properties. J. Polym. Sci. 2024, 62, 155–164. 10.1002/pol.20230283.

[ref33] Hu M.; Ling Z.; Ren X. Extracellular matrix dynamics: tracking in biological systems and their implications. J. Biol. Eng. 2022, 16, 13 10.1186/s13036-022-00292-x. 35637526 PMC9153193

[ref34] Fujishige S.; Kubota K.; Ando I. Phase transition of aqueous solutions of poly(N-isopropylacrylamide) and poly(N-isopropylmethacrylamide). J. Phys. Chem. 1989, 93, 3311–3313. 10.1021/j100345a085.

[ref35] Fernández-Castaño Romera M.; et al. Mimicking Active Biopolymer Networks with a Synthetic Hydrogel. J. Am. Chem. Soc. 2019, 141, 1989–1997. 10.1021/jacs.8b10659. 30636412 PMC6367683

[ref36] De Almeida P.; et al. Cytoskeletal stiffening in synthetic hydrogels. Nat. Commun. 2019, 10, 609 10.1038/s41467-019-08569-4. 30723211 PMC6363731

[ref37] Greenspan P.; Fowler S. D. Spectrofluorometric studies of the lipid probe, nile red. J. Lipid Res. 1985, 26, 781–789. 10.1016/S0022-2275(20)34307-8. 4031658

[ref38] Sackett D. L.; Wolff J. Nile red as a polarity-sensitive fluorescent probe of hydrophobic protein surfaces. Anal. Biochem. 1987, 167, 228–234. 10.1016/0003-2697(87)90157-6. 3442318

[ref39] Börgardts M.; et al. Synthesis and optical properties of covalently bound Nile Red in mesoporous silica hybrids-comparison of dye distribution of materials prepared by facile grafting and by co-condensation routes. RSC Adv. 2016, 6, 6209–6222. 10.1039/C5RA22736D.

[ref40] Boldon L.; Laliberte F.; Liu L. Review of the fundamental theories behind small angle X-ray scattering, molecular dynamics simulations, and relevant integrated application. Nano Rev. 2015, 6, 25661 10.3402/nano.v6.25661. 25721341 PMC4342503

[ref41] Burla F.; Tauber J.; Dussi S.; van der Gucht J.; Koenderink G. H. Stress management in composite biopolymer networks. Nat. Phys. 2019, 15, 549–553. 10.1038/s41567-019-0443-6.

[ref42] Zhu Z.; Yang C. J. Hydrogel droplet microfluidics for high-throughput single molecule/cell analysis. Acc. Chem. Res. 2017, 50, 22–31. 10.1021/acs.accounts.6b00370. 28029779

[ref43] Hasturk O.; Kaplan D. L. Cell armor for protection against environmental stress: Advances, challenges and applications in micro-and nanoencapsulation of mammalian cells. Acta Biomater. 2019, 95, 3–31. 10.1016/j.actbio.2018.11.040. 30481608 PMC6534491

[ref44] Goy C. B.; Chaile R. E.; Madrid R. E. Microfluidics and hydrogel: A powerful combination. React. Funct. Polym. 2019, 145, 104314 10.1016/j.reactfunctpolym.2019.104314.

[ref45] Tiemeijer B. M.; et al. Probing Single-Cell Macrophage Polarization and Heterogeneity Using Thermo-Reversible Hydrogels in Droplet-Based Microfluidics. Front. Bioeng. Biotechnol. 2021, 9, 715408 10.3389/fbioe.2021.715408. 34722475 PMC8552120

[ref46] Sinha N.; Subedi N.; Wimmers F.; Soennichsen M.; Tel J. A Pipette-Tip Based Method for Seeding Cells to Droplet Microfluidic Platforms. J. Vis. Exp. 2019, e57848 10.3791/57848. 30799837

[ref47] Rijns L.; et al. The Importance of Effective Ligand Concentration to Direct Epithelial Cell Polarity in Dynamic Hydrogels. Adv. Mater. 2023, 2300873 10.1002/adma.202300873. 37264535

[ref48] Hersel U.; Dahmen C.; Kessler H. RGD modified polymers: biomaterials for stimulated cell adhesion and beyond. Biomaterials 2003, 24, 4385–4415. 10.1016/S0142-9612(03)00343-0. 12922151

