# Supplementary material for: Tuning Structural Organization via Molecular Design and Hierarchical Assembly to Develop Supramolecular Thermoresponsive Hydrogels
Source: Macromolecules. 2024 Jul 3;57(14):6606–15. doi: 10.1021/acs.macromol.4c00567 (PMC11270986; doi:10.1021/acs.macromol.4c00567)
Supplement: Supplementary file 1 — ma4c00567_si_001.pdf [file ma4c00567_si_001.pdf]

Supporting Information

Tuning structural organization via molecular design and hierarchical assembly to develop supramolecular thermo-responsive hydrogels

Dan Jing Wu,[1,2,3] Martin G. T. A. Rutten,[2,3] Jingyi Huang,[1,2,3] Maaike J. G. Schotman,[2,3] Johnick F. van Sprang,[1,2,3] Bart M. Tiemeijer,[2,5] Gijs M. ter Huurne,[2,4] Sjors P. W. Wijnands,[2,3] Mani Diba,[2,3,6] Patricia Y. W. Dankers[1,2,3,*]

[1] Laboratory for Cell and Tissue Engineering, Department of Biomedical Engineering, Eindhoven University of Technology, Eindhoven, PO Box 513, 5600 MB Eindhoven, The Netherlands.

[2] Institute for Complex Molecular Systems, Eindhoven University of Technology, Eindhoven, PO Box 513, 5600 MB The Netherlands.

[3] Laboratory of Chemical Biology, Department of Biomedical Engineering, Eindhoven University of Technology, Eindhoven, PO Box 513, 5600 MB The Netherlands.

[4] Laboratory of Macromolecular and Organic Chemistry, Department of Chemical Engineering and Chemistry, Eindhoven University of Technology, Eindhoven, PO Box 513, 5600 MB The Netherlands

[5] Laboratory of Immunoengineering, Department of Biomedical Engineering, Eindhoven University of Technology, Eindhoven, 5600 MB, the Netherlands

[6] Department of Dentistry-Regenerative Biomaterials, Research Institute for Medical Innovation, Radboud University Medical Center, Nijmegen, 6525EX, the Netherlands

[*] Email corresponding author: p.y.w.dankers@tue.nl

Table of contents

1. Materials ... 3
2. Methods... 3
2.1. Cryogenic Transmission Electron Microscopy (cryo-TEM) ... 3
2.2. Total internal reflection fluorescence (TIRF)... 3
2.3. Nile red fluorescence measurements ... 4
2.4. Static light scattering (SLS)... 4
2.5. Rheology ... 5
2.6. Small angle X-ray scattering ... 5
3. Synthesis procedure ... 6
4. Cell culture... 19
4.1. Microfluidic device production ... 19
4.2. Cells encapsulation in hydrogel droplets... 19
5. Additional results ... 21
5.1. Cryogenic Transmission Electron Microscopy (cryo-TEM) ... 21
5.2. Total internal reflection fluorescence (TIRF)... 22
5.3. Nile red fluorescence measurements ... 23
5.4. Rheology ... 24
5.5. Small angle X-ray scattering ... 25
6. References ... 26

1. Materials

All compounds were used as received, unless stated otherwise. Poly(N-isopropylacrylamide), amine terminated (M_n~2.5, 5.5 kDa) was purchased from Sigma Aldrich. Rink amide MBHA resin, Fmoc protected amino acids and 1-(Bis(dimethylamino)methylene)-1H-1,2,3-triazolo(4,5-b)pyridinium 3-oxide hexafluorophosphate (HATU) were purchased from Novabiochem. The solvents N-methyl-2-pyrrolidone (NMP) and Acetonitrile (ACN) were purchased from Actu-All chemicals. The compounds HATU, 2-(1H-benzotriazol-1-yl)-1,1,3,3-tetramethyluronium hexafluorophosphate (HBTU), N,N-diisopropylethylamine (DIPEA), trifluoroacetic acid (TFA), piperidine, dichloromethane (DCM), and dimethylformamide (DMF) were purchased from Biosolve. Water was purified on an EMD Milipore Milli-Q Integral Water Purification System. The UPy-OMe (**5**) and the UPy-hexyl-isocyanate monomers were synthesized by SyMO-Chem BV, Eindhoven, The Netherlands.

2. Methods

2.1. Cryogenic Transmission Electron Microscopy (cryo-TEM)

For cryo-TEM measurements quantifoil carbon film grids were used (Cu200 mesh, R2/1). Prior to sample addition, grids were surface plasma treated (at 5 mA for 40s) using a Cressington 208 carbon coater. Using an automated vitrification robot (FEI VitrobotTM Mark III), 3 μL sample was applied to the grids and excess sample was removed by blotting, using filter paper for 3 s at –3 mm. The thin film formed was vitrified by plunging the grid into liquid ethane just above its freezing point. On a FEI-Titan TEM equipped with a field emission gun operating at 300 kV the samples were examined. Post-GIF (Gatan imaging filter) 2x2 Gatan CCD camera was used for recording of the images. Micrographs were taken at low dose conditions, using a defocus setting of 10 μm at 25k magnification, or defocus setting of 40 μm at 6.5k magnification.

2.2. Total internal reflection fluorescence (TIRF)

Total internal reflection fluorescence (TIRF) images and videos were acquired with a Nikon N-STORM system. Nile red was excited using a 561 nm laser. Fluorescence was collected by means of a Nikon 100x, 1.4NA oil immersion objective and passed through a quad-band pass dichroic filter (97335 Nikon). All material was recorded with an EMCCD camera (ixon3, Andor, pixel size 0.17μm). Samples were flown in a chamber between glass microscope coverslips (Menzel-Gläser, No. 1, 24x24 mm) and glass slides which were separated by double-sided tape.

2.3. Nile red fluorescence measurements

The emission scans were performed with a 5% Nile red in 200 µM UPy-C_n-PNIPAM monomer solution (in Milli-Q). Each time upon increasing the temperature, the sample was equilibrated for 10 min. NR was excited at 550 nm, and the emission intensity was recorded from 565-800 nm. Maximum emission intensity values are plotted versus wavelength.

2.4. Static light scattering (SLS)

Light scattering experiments were performed on an ALV Compact Goniometer System (CGS-3) Multi-Detector (MD-4) which was equipped with ALV-7004 Digital Multiple Tau Real Time Correlator and a 532 nm laser. The temperature was regulated at 20.0 ± 0.2 °C using a Lauda RM6-S refrigerated circulating bath. The light scattering intensity was recorded at scattering angles (θ ranging from 30° and 150° in steps of 5° with an average over 6 runs of 15 seconds per angle. The samples were filtered using polyvinylidene fluoride (PVDF) filter with pores of 0.2 µm and measured in disposable tubes of glass with an outer diameter of 10 mm. The absolute scattering intensity of Rayleigh ratio (R_θ) was computed according to equation 1:

$$R_\theta = \frac{I_s - I_{sol}}{I_{tol}} \cdot \left(\frac{n_{sol}}{n_{tol}}\right)^2 \cdot R_{\theta,toluene} \qquad (1)$$

With I_s, I_{sol} and I_{tol} being the measured scattering intensities of the sample, solvent and toluene. The refractive indices n_{sol} (= 1.33) and n_{tol} (= 1.49), and Rayleigh ratio of toluene ($R_{\theta,\ toluene}$ = $2.1 \cdot 10^{-5}$ cm^{-1} at 532 nm).[2] Intensity cross-correlation function ($g^{(2)}(\tau)$) were measured at scattering vectors q, defined as in equation 2:

$$q = \frac{4\pi \cdot n_{sol}}{\lambda} \cdot sin\left(\frac{\theta}{2}\right) \qquad (2)$$

Moreover, the electric field cross-correlation function ($g^{(1)}(\tau)$) were calculated according to the Siegert equition (in Equation 3) and analyzed with the CONTIN algorithm to determine distributions of the relaxation times (τ_D):

$$g^{(1)}(\tau) = \sqrt{\frac{(g^{(2)}(\tau)-1)}{\beta}} \qquad (3)$$

The apparent diffusion constant D_{app} was determined from the slope of the obtained relaxation times τ_D versus q^2. Furthermore, the hydrodynamic radius (R_H) was determined using the

Stokes-Einstein relationship (equation 4) This formula contains the Boltzmann constant (k_B), temperature (T), viscosity (η) and diffusion coefficient (D):

$$R_H = \frac{k_B \cdot T}{6\pi \cdot \eta \cdot D} \qquad (4)$$

2.5. Rheology

Rheological measurements were carried out on a TA Instruments Dynamic Hybrid Rheometer 3 in a 20 mm aluminum cone-plate (2.007°) geometry with a truncation gap of 56 µm or on a Anton Paar MCR 501, equipped with a 25 mm cone-plate (1.006°) geometry with a truncation gap of 49 µm. A solvent trap was used to minimize sample drying. Samples were loaded at 20 °C and allowed to equilibrate for a short period. Temperature dependent measurements were carried out at a ramp rate of 1 °C/min while the complex modulus G* was measured by applying an oscillating deformation of amplitude γ = 1% at frequency ω = 1 rad/s.

2.6. Small angle X-ray scattering

Small-angle X-ray scattering (SAXS) profiles were recorded on SAXLAB GANESHA 300 XL SAXS equipped with a GeniX 3D Cu Ultra Low Divergence micro focus sealed tube source producing Xrays with a wavelength λ = 1.54 Å at a flux of 1×108 ph s-1 and a Pilatus 300 K silicon pixel detector with 487 × 619 pixels of 172 × 172 μm^2 in size placed a three sample-to-detector distances of 113, 713, and 1513 mm respectively to cover a q-range of 0.07 ≤ q ≤ 3.0 nm^{-1} with q = 4π/λ(sin θ/2). Silver behenate was used for calibration of the beam center as well as the q-range. Samples were measured within 2 mm quartz capillaries (Hilgenberg GmbH, Germany). The two-dimensional SAXS patterns were brought to an absolute intensity scale using the calibrated detector response function, known sample-to-detector distance, measured incident and transmitted beam intensities, and azimuthally averaged to obtain one-dimensional SAXS profiles. The scattering curves of the fibers were obtained by subtraction of the scattering contribution of the solvent and quartz cell. Temperature was controlled at 20 and 40 °C using a PT100 sensor and a Julabo temperature controller.

3. Synthesis procedure

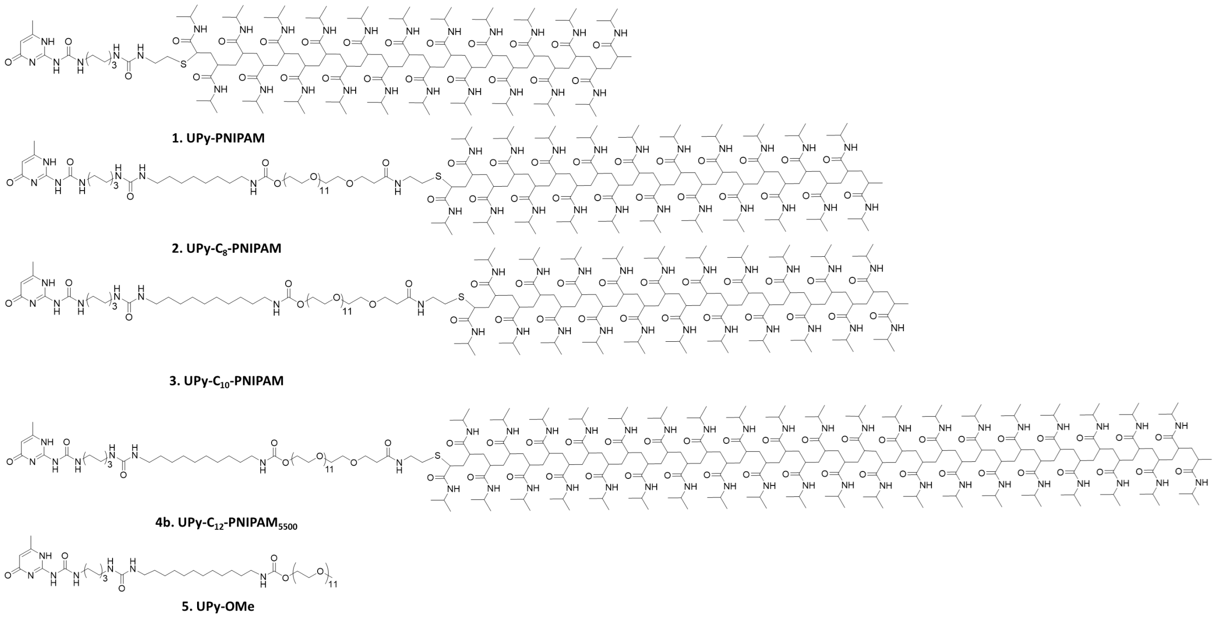

Scheme S1 Schematic overview of supramolecular monomers. A. *Chemical structures of* ***UPy-PNIPAM (1), UPy-C_8-PNIPAM (2), UPy-C_{10}-PNIPAM (3), UPy-C_{12}-$PNIPAM_{5500}$ (4b)*** *and* ***UPy-OMe (5)****.*

*Synthesis of UPy -PNIPAM (**1**).* UPy-hexyl-isocyanate (UPy-NCO, 3.3 mmol, 1 eq) and 1.2 eq. NH_2-PNIPAM (M_n 2500 g/mol) were dissolved in dimethylformamide to a solution of anhydrous tetrahydrofuran (THF) as solvent and the mixture was activated using 1.2 eq. HATU and 3 eq. triethylamine (TEA) stirred overnight. Ninhydrin staining was used to confirm that the primary amines had reacted. The access of NH_2-PNIPAM was removed using isocyanate functionalized polystyrene resin by stirring for 3 hours at RT . The solution was filtered and precipitated in DCM/hexane and dried overnight. The compound was dried overnight, resulting in a white solid (1.15 g, 98% yield). ^{1}H NMR (400 MHz, Chloroform-*d*) δ 13.06 (s, 0H), 11.76 (s, 1H), 9.98 (s, 1H), 9.05 (s, 1H), 6.97 – 5.87 (m, 14H), 5.77 (s, 1H), 5.70 – 5.07 (m, 0H), 3.94 (d, *J* = 15.0 Hz, 31H), 3.71 – 3.61 (m, 1H), 3.61 – 2.95 (m, 5H), 2.39 (s, 30H), 2.29 – 1.39 (m, 58H), 1.39 – 0.24 (m, 209H). NMR: M_w 16690 g/mol, M_n 6338 g/mol, Đ = 2.6.

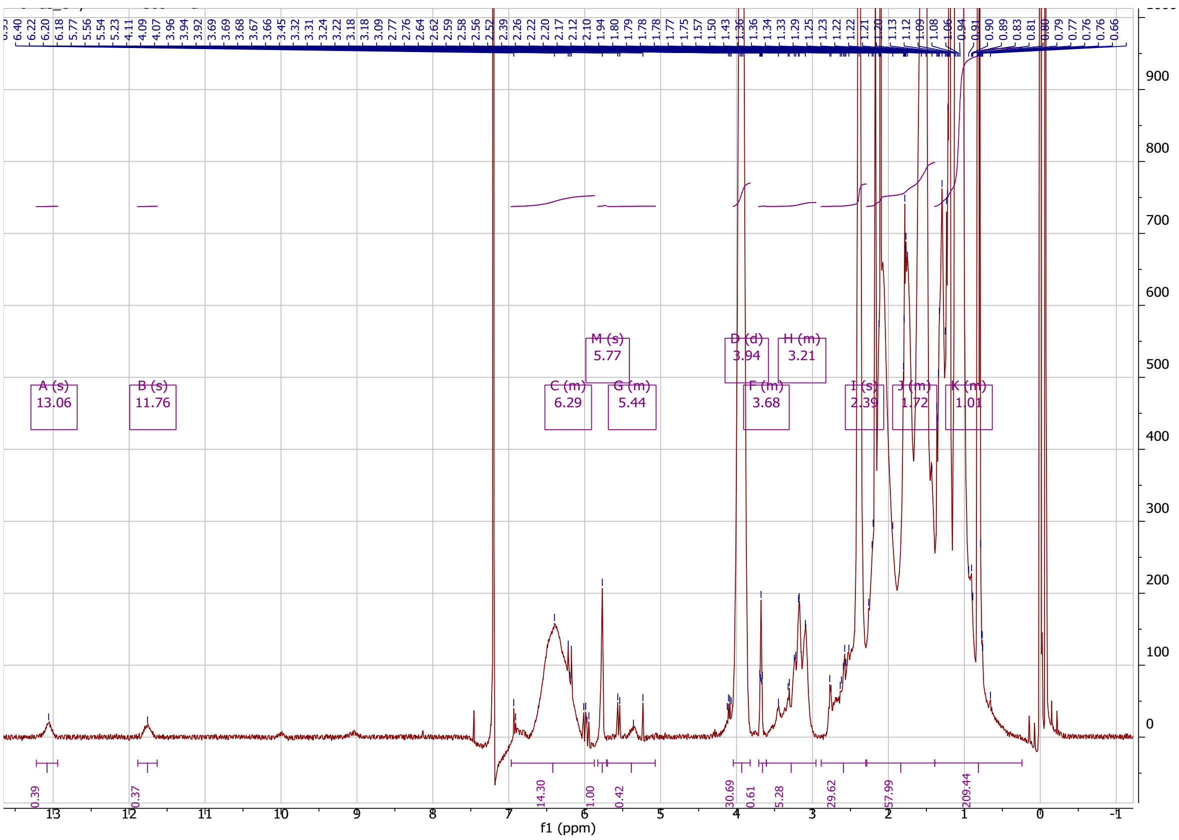

Synthesis of UPy-C_n-COOH

Synthesis of compound (7). Benzyl carbonate (7.15 g, 31 mmol) was dissolved in 10 mL ethanol and was added dropwise to a solution of 1,8-diaminooctane (**6**) (4.9 g, 33.8 mmol) in 25 mL ethanol. The resulting mixture was stirred overnight at reflux. After cooling to room temperature, 50 mL of ethanol was added, resulting in a precipitate that was removed by filtration and washed with 100 mL of ethanol. The ethanol was subsequently concentrated to approximately 10 mL and stirred with aqueous HCl (450 mL, 1M) for 30 minutes. The resulting salt was collected by filtration, washed with 1 M HCl, and stirred for 16 hours in aqueous HCl (250 mL, 1M). The solid was collected by filtration and washed with diethyl ether (2x 100 mL) and dichloromethane (3x 100 mL). The resulting residue was dissolved in 200 mL of dichloromethane and 50 mL of ethanol. This solution was washed twice with 50 mL of a 1 M solution of sodium hydroxide. The aqueous phase was extracted twice with 50 mL dichloromethane and once with 50 mL of chloroform containing 10 % methanol. The combined organic layers were dried over Na_2SO_4 and concentrated in vacuo to afford the crude product, which was purified by column chromatography using silica, eluting with dichloromethane containing 10–15% methanol and 1–2% triethylamine yielding the pure product (3.9 g, 45 %). ^{1}H NMR (400 MHz, $CDCl_3$): δ = 1.15 (s, 2H), 1.26 (m, 8H), 1.45 (m, 4H), 2.65 (t, 2H), 3.18 (q, 2H), 5.08 (s, 2H), 7.37 (m, 5H). ^{13}C NMR (400 MHz, $CDCl_3$): δ = 26.58, 26.68, 29.11, 29.64,

29.37, 29.40, 29.68, 32.95, 40.80, 41.84, 66.38, 75.60, 75.62, 127.79, 127.88, 128.32, 136.50, 156.77. RP-LCMS: calc. m/z 278.2, found: $[M+H]^+$ 279.4.

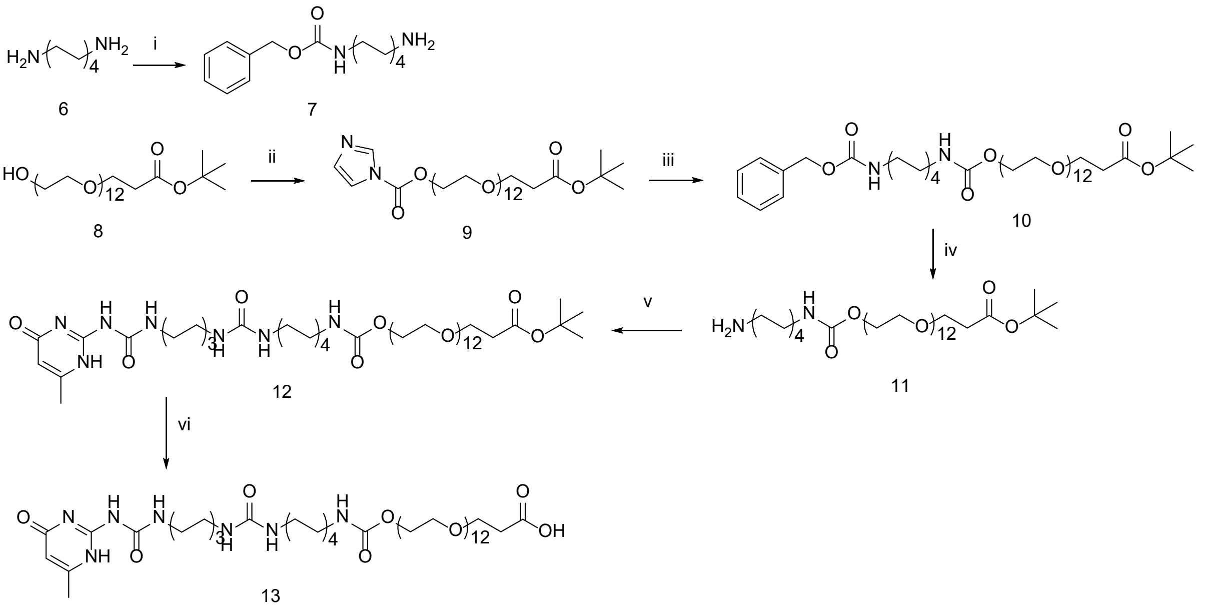

Scheme S2 Synthesis of UPy-building block 13. *i) BPC, EtOH, reflux at 65 °C, o.n., 45 %; ii) CDI, CHCl3, RT, 24 h, quantitative; iii) CbzNHC$_{12}$H$_{24}$, CHCl$_3$, 65 °C, 24 h, 67%; iv) 5 % Pd/C, H$_2$, EtOH, RT, 20 h, 95 %; vi) UPy-C$_6$-NCO, DIPEA, CHCl$_3$, 65 °C, 1 h, 85 %; vii) TFA, DCM, RT, 3 h, 88 %.*

*Synthesis of compound (**10**) via (**9**).* OEG$_{12}$-tBu (**8**) (1 g, 1.48 mmol) was dissolved in 5 mL chloroform and was added dropwise to a solution of N,N-carbonyldiimidazole (0.264 g, 1.63 mmol) in 5 mL chloroform. The reaction mixture was stirred at room temperature for 24 hours. Amine **7** (0.453 g, 1.63 mmol) was added to this reaction mixture and stirring continued for 2 hours at 65 °C, affording a turbid solution. The reaction mixture was cooled to room temperature and the precipitate was removed by filtration. Analysis by NMR revealed incomplete conversion to the carbamate. Additional amine (**7**) (0.18 g, 0.65 mmol) was added to the reaction mixture, which was heated at reflux for another 24 hours to afford full conversion. The reaction mixture was then diluted with 40 mL of chloroform and washed with a 0.5 M aqueous citric acid solution (2x 20 mL) and a saturated sodium chloride solution (20 mL), drying over $MgSO_4$ and concentrated in vacuo. The crude product was purified by silica column chromatography using chloroform containing 2–5% of methanol to afford the pure product (0.97 g, 67%). ^1H NMR (400 MHz, $CDCl_3$): δ = 1.26 (m, 8H), 1.47 (m, 13H), 2.51 (t, 2H), 3.18 (m, 4H), 3.65 (m, 48H), 4.21 (t, 2H), 5.08 (s, 2H), 7.37 (m, 5H). ^{13}C NMR (400 MHz, $CDCl_3$): δ = 170.86, 156.36, 136.66, 128.46, 128.07, 128.02, 80.46, 70.59, 70.54, 70.48, 70.47,

70.33, 69.67, 66.87, 66.51, 63.76, 41.09, 41.02, 36.24, 29.93, 29.47, 29.22, 28.08, 26.71. RP-LCMS: calc. m/z 978.6, found: $[M+H]^+$ 979.5.

Synthesis of compound (11). 0.97 g of tBu-OEG$_{12}$-C$_8$-diamine-Z (**10**) was dissolved in 25 mL ethanol in a round bottom flask provided with one stopcock attached to an argon / vacuum line and one stopcock attached to a balloon filled with H_2 After addition of 90 mg Pd/C (5% Pd, Degussa type), the system was purged and brought to a hydrogen atmosphere. The reaction mixture was stirred for 20 hours at room temperature, purging and refilling the balloon as necessary, leading to full conversion. The reaction mixture was filtered over Celite and evaporated to dryness affording the pure product (0.8 g, 95%). ^{1}H NMR (400 MHz, $CDCl_3$): δ = 1.31 (m, 8H), 1.45 (s, 9H), 1.50 (m, 4H), 1.76 (t, 2H), 2.51 (t, 2H), 2.94 (t, 2H), 3.18 (q, 4H), 3.65 (m, 48H), 4.21 (t, 2H), 5.40 (t, 1H). ^{13}C NMR (400 MHz, $CDCl_3$): δ = 170.85, 156.38, 80.45, 70.48, 70.31, 69.81, 66.85, 63.60, 41.00, 40.13, 36.23, 29.83, 29.30, 29.27, 29.23, 29.09, 28.93, 28.06, 27.90, 26.64, 26.54. RP-LCMS: calc. m/z 844.5, found: $[M+H]^+$ 845.8.

Synthesis of compound (12). NH$_2$-C$_{10}$-OEG$_{12}$-tBu (**11**) (0.8 g, 0.95 mmol), UPy-C$_6$-NCO (0.28 g, 0.95 mmol) and N,N-diisopropylethylamine (0.18 mL, 1.01 mmol) were stirred in 15 mL chloroform at 65 °C. After 1 hour no isocyanate absorption was observed with FT-IR, 2 mL ethanol was added and the mixture was filtered over Celite. The filtrate was concentrated and dissolved in 8 mL chloroform and 2 mL of ethanol and precipitated in 100 mL pentane. The product was collected by filtration and washed with pentane. Drying yielded the pure product (0.92 g, 85%). ^{1}H NMR (400 MHz, $CDCl_3$): δ = 1.4 (m, 35H(29H)), 2.22 (s, 3H), 2.51 (t, 2H), 3.20 (m, 8H), 3.65 (m, 48H), 4.21 (t, 2H), 4.63 (s, NH), 4.85 (s, NH), 4.95 (s, NH), 5.83 (s, 1H), 10.05 (s, NH), 11.85 (s, NH), 13.18 (s, NH). RP-LCMS: calc. m/z 1137.7, found: $[M+H+Na]^{2+}$ 581.0, $[M+Na]^+$ 1160.6.

Synthesis of compound (13). UPy-C$_8$-OEG$_{12}$-tBu (**12**) was dissolved in 20 mL dichloromethane and 5 mL trifluoro acetic acid was added. After stirring for 3 hours at room temperature the reaction was concentrated and co-evaporated three times with toluene. The crude product was dissolved in 6 mL chloroform and 0.5 mL ethanol and precipitated in 100 mL diisopropyl ether. The product was collected by filtration and washed with diisopropyl ether. Drying yielded 0.77 g (88%) of the pure product. ^{1}H NMR (400 MHz, $CDCl_3$): δ = 1.4 (m, 20H), 2.25 (s, 3H), 2.62 (t, 2H), 3.20 (m, 5H), 3.65 (m, 48H), 4.21 (t, 2H), 4.95 (s,3 NH), 5.83 (s, 1H), 10.05 (s, NH), 11.83 (s, NH), 13.16 (s, NH). ^{13}C NMR (400 MHz, $CDCl_3$): δ = 187.26, 187.24, 156.48, 139.45,

106.52, 75.60, 70.53, 70.29, 69.67, 66.70, 63.79, 40.59, 39.55, 35.05, 30.15, 29.90, 29.45, 29.28, 29.20, 26.87, 26.69, 18.95. RP-LCMS: calc. m/z 1081.6, found: $[M+H]^+$ 1082.58, $[M+2H]^{2+}$ 542.0.

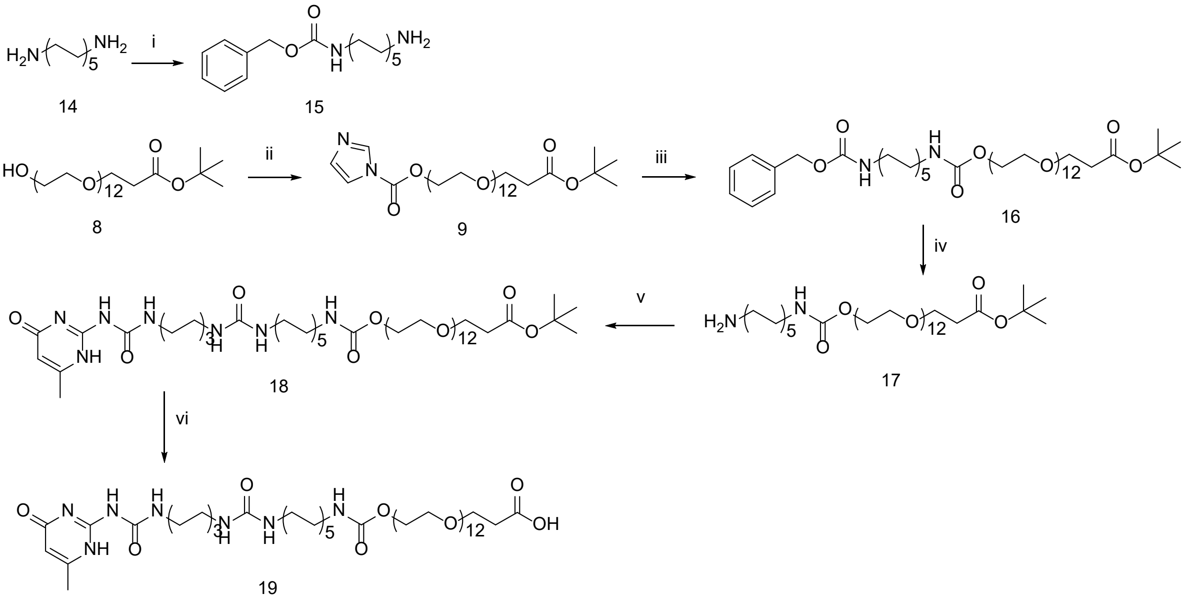

Scheme S3 Synthesis of UPy-building block 19. *i) BPC, EtOH, reflux at 65 °C, o.n., 51 %; ii) CDI, CHCl3, RT, 24 h, quantitative; iii)* $CbzNHC_{12}H_{24}$*,* $CHCl_3$*, 65 °C, 24 h, 65%; iv) 5 % Pd/C,* H_2*, EtOH, RT, 20 h, 93 %; vi) UPy-*C_6*-NCO, DIPEA,* $CHCl_3$*, 65 °C, 1 h, 87 %; vii) TFA, DCM, RT, 3 h, 89 %.*

*Synthesis of compound (**15**).* Benzyl carbonate (6.69 g, 29 mmol) was dissolved in 10 mL ethanol and was added dropwise to a solution of 1,10-diaminodecane (**14**) (5 g, 29 mmol) in 25 mL ethanol. The resulting mixture was stirred overnight at reflux. After cooling to room temperature, 50 mL of ethanol was added, resulting in a precipitate that was removed by filtration and washed with 100 mL of ethanol. The ethanol was subsequently concentrated to approximately 10 mL and stirred with aqueous HCl (450 mL, 1M) for 30 minutes. The resulting salt was collected by filtration, washed with 1 M HCl, and stirred for 16 hours in aqueous HCl (250 mL, 1M). The solid was collected by filtration and washed with diethyl ether (2x 100 mL) and dichloromethane (3x 100 mL). The resulting residue was dissolved in 200 mL of dichloromethane and 50 mL of ethanol. This solution was washed twice with 50 mL of a 1 M solution of sodium hydroxide. The aqueous phase was extracted twice with 50 mL dichloromethane and once with 50 mL of chloroform containing 10 % methanol. The combined organic layers were dried over Na_2SO_4 and concentrated in vacuo to afford the crude product, which was purified by column chromatography using silica, eluting with dichloromethane containing 10–15% methanol and 1–2% triethylamine yielding the pure product (4.5 g, 51 %). ^1H NMR (400 MHz, $CDCl_3$): δ = 1.15 (s, 2H), 1.26 (m, 12H), 1.45 (m, 4H), 2.65 (t, 2H), 3.18

(q, 2H), 5.08 (s, 2H), 7.37 (m, 5H).^{13}C NMR (400 MHz, $CDCl_3$): δ = 26.58, 26.68, 29.12, 29.64, 29.35, 29.43, 29.68, 32.95, 40.81, 41.84, 66.37, 75.60, 75.62, 127.79, 127.86, 128.32, 136.50, 156.75. RP-LCMS: calc. m/z 306.2, found: $[M+H]^+$ 307.5.

*Synthesis of compound (**16**) via (**9**).* OEG_{12}-tBu (**8**) (1 g, 1.48 mmol) was dissolved in 5 mL chloroform and was added dropwise to a solution of N,N-carbonyldiimidazole (0.264 g, 1.63 mmol) in 5 mL chloroform. The reaction mixture was stirred at room temperature for 24 hours. Amine **15** (0.499 g, 1.63 mmol) was added to this reaction mixture and stirring was continued for 2 hours at 65 °C, affording a turbid solution. The reaction mixture was cooled to room temperature and the precipitate was removed by filtration. Analysis by NMR revealed incomplete conversion to the carbamate. Additional amine **15** (0.2 g, 0.65 mmol) was added to the reaction mixture, which was heated at reflux for another 24 hours to afford full conversion. The reaction mixture was then diluted with 40 mL of chloroform and washed with a 0.5 M aqueous citric acid solution (2x 20 mL) and a saturated sodium chloride solution (20 mL), drying over $MgSO_4$ and concentrated in vacuo. The crude product was purified by silica column chromatography using chloroform containing 2–5% of methanol to afford the pure product (0.96 g, 65%). ^{1}H NMR (400 MHz, $CDCl_3$): δ = 1.26 (m, 12H), 1.47 (m, 13H), 2.51 (t, 2H), 3.18 (m, 4H), 3.65 (m, 48H), 4.21 (t, 2H), 5.08 (s, 2H), 7.37 (m, 5H). ^{13}C NMR (400 MHz, $CDCl_3$): δ = 170.86, 156.36, 136.66, 128.46, 128.07, 128.02, 80.47, 70.59, 70.54, 70.48, 70.44, 70.33, 69.67, 66.87, 66.52, 63.76, 41.09, 41.05, 36.25, 29.93, 29.47, 29.22, 28.07, 26.71. RP-LCMS: calc. m/z 1006.6, found: $[M+H]^+$ 1007.5.

*Synthesis of compound (**17**).* 0.96 g of tBu-OEG_{12}-C_{10}-diamine-Z (**16**) was dissolved in 25 mL ethanol in a round bottom flask provided with one stopcock attached to an argon / vacuum line and one stopcock attached to a balloon filled with H_2. After addition of 90 mg Pd/C (5% Pd, Degussa type), the system was purged and brought to a hydrogen atmosphere. The reaction mixture was stirred for 20 hours at room temperature, purging and refilling the balloon as necessary, leading to full conversion. The reaction mixture was filtered over Celite and evaporated to dryness affording the pure product (0.77 g, 93%). ^{1}H NMR (400 MHz, $CDCl_3$): δ = 1.31 (m, 12H), 1.45 (s, 9H), 1.50 (m, 4H), 1.76 (t, 2H), 2.51 (t, 2H), 2.94 (t, 2H), 3.18 (q, 4H), 3.65 (m, 48H), 4.21 (t, 2H), 5.40 (t, 1H). ^{13}C NMR (400 MHz, $CDCl_3$): δ = 170.84, 156.36, 80.45, 70.49, 70.30, 69.81, 66.85, 63.60, 41.00, 40.14, 36.23, 29.86, 29.30, 29.27, 29.28, 29.09, 28.94, 28.06, 27.92, 26.64, 26.55. RP-LCMS: calc. m/z 872.6, found: $[M+H]^+$ 873.8.

*Synthesis of compound (**18**).* NH_2-C_{10}-OEG_{12}-tBu (**17**) (0.77 g, 0.88 mmol), UPy-C_6-NCO (0.26 g, 0.88 mmol) and N,N-diisopropylethylamine (0.18 mL, 1.01 mmol) were stirred in 15 mL chloroform at 65 °C. After 1 hour no isocyanate absorption was observed with FT-IR, 2 mL ethanol was added and the mixture was filtered over Celite. The filtrate was concentrated and dissolved in 8 mL chloroform and 2 mL of ethanol and precipitated in 100 mL pentane. The product was collected by filtration and washed with pentane. Drying yielded of the pure product (0.89 g, 87%). ^{1}H NMR (400 MHz, $CDCl_3$): δ = 1.4 (m, 39H(33H)), 2.22 (s, 3H), 2.51 (t, 2H), 3.20 (m, 8H), 3.65 (m, 48H), 4.21 (t, 2H), 4.63 (s, NH), 4.85 (s, NH), 4.95 (s, NH), 5.83 (s, 1H), 10.05 (s, NH), 11.85 (s, NH), 13.18 (s, NH). RP-LCMS: calc. m/z 1165.7, found: $[M+H+Na]^{2+}$ 583.83, $[M+H]^{+}$ 1166.8.

*Synthesis of compound (**19**).* UPy-C_{10}-OEG_{12}-tBu (**18**) was dissolved in 20 mL dichloromethane and 5 mL trifluoro acetic acid was added. After stirring for 3 hours at room temperature the reaction was concentrated and co-evaporated three times with toluene. The crude product was dissolved in 6 mL chloroform and 0.5 mL ethanol and precipitated in 100 mL diisopropyl ether. The product was collected by filtration and washed with diisopropyl ether. Drying yielded 0.76 g (89%) of the pure product. ^{1}H NMR (400 MHz, $CDCl_3$): δ = 1.4 (m, 24H), 2.25 (s, 3H), 2.62 (t, 2H), 3.20 (m, 5H), 3.65 (m, 48H), 4.21 (t, 2H), 4.95 (s,3 NH), 5.83 (s, 1H), 10.05 (s, NH), 11.83 (s, NH), 13.16 (s, NH). ^{13}C NMR (400 MHz, $CDCl_3$): δ = 187.23, 187.24, 156.48, 139.46, 106.52, 75.60, 70.53, 70.29, 69.67, 66.70, 63.79, 40.59, 39.55, 35.05, 30.15, 29.91, 29.45, 29.28, 29.21, 26.86, 26.70, 18.95. RP-LCMS: calc. m/z 1109.67, found: $[M+H]^{+}$ 1110.67.

*The UPy-OEG_{12}-COOH (**20**) precursor was synthesized as previously reported.*[1]

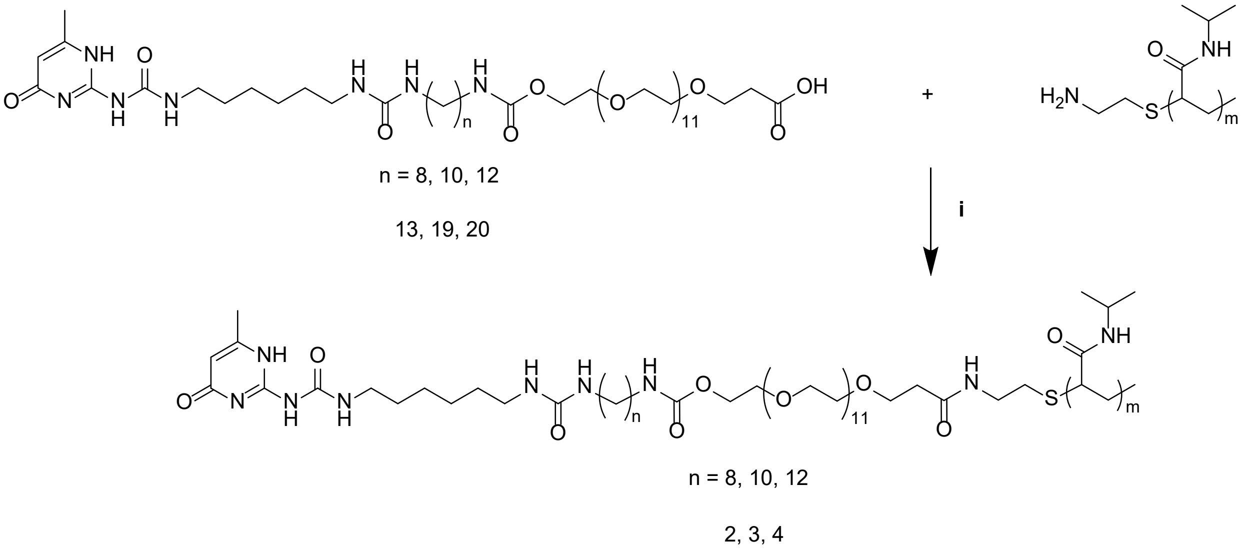

Scheme S4 Synthesis of UPy-C$_n$-PNIPAM monomers (n = 8, 10, 12). *UPy-modified PNIPAM monomers **2**, **3**, **4** with different alkyl spacers were synthesized using amide coupling $M_{n,PNIPAM}$ ~ 2.5 or 5.5 kg/mol. **i**. HATU, DMF/$CHCl_3$, n-methylmorpholine, overnight, 60 °C.*

*Synthesis of **UPy-C$_8$-PNIPAM (2)***: UPy-C$_8$-carboxylic acid (**13**) (3.3 mmol, eq) and 1.2 eq. NH_2-PNIPAM (Mn 2500 g/mol) were dissolved in dimethylformamide (DMF) and chloroform ($CHCl_3$) and the mixture was activated using 1.2 eq HATU, 3 eq. methyl-morpholine and stirred at 60 °C overnight. Ninhydrin staining was used to confirm that the primary amines had reacted. The unreacted UPy-hexyl-COOH was removed via extraction with 2-isopropyl ether. And the access of NH_2-PNIPAM was removed using a methyl isocyanate bound to polystyrene resin and methyl-morpholine by stirring for 3 hours at RT. The solution was filtered and dried overnight, resulting in a white solid with 97,6 % yield. ^{1}H NMR (400 MHz, Chloroform-*d*) δ = 13.12 (s, 1H), 11.82 (s, 1H), 10.07 (s, 1H), 8.73 (s, 1H), 8.26 (s, 1H), 7.17 – 5.96 (m, 72H), 5.84 (s, 1H), 5.25 – 4.53 (m, 1H), 4.20 (t, *J* = 4.7 Hz, 2H), 4.03 (s, 14H), 3.65 (d, *J* = 1.9 Hz, 46H), 3.48 – 3.31 (m, 1H), 3.31 – 2.40 (m, 65H), 2.40 – 1.23 (m, 47H), 1.14 (d, *J* = 5.4 Hz, 123H). GPC: M_w 9792 g/mol, M_n 2880 g/mol, Đ = 3.4

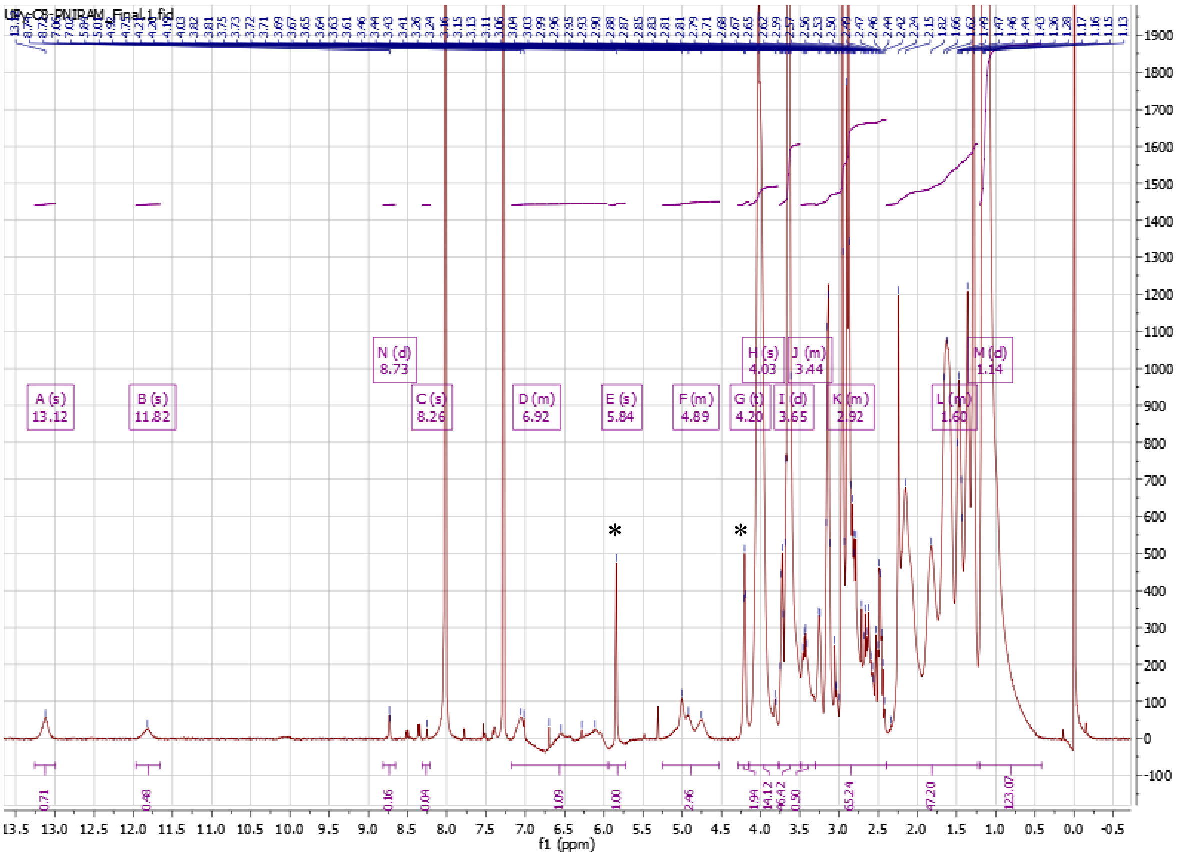

*Synthesis of **UPy-C$_{10}$-PNIPAM (3)***: UPy-C$_{10}$-carboxylic acid (**19**) (3.3 mmol, eq) and 1.2 eq. NH_2-PNIPAM (Mn 2500 g/mol) were dissolved in dimethylformamide (DMF) and chloroform ($CHCl_3$) and the mixture was activated using 1.2 eq HATU, 3 eq. methyl-morpholine and stirred at 60 °C overnight. Ninhydrin staining was used to confirm that the primary amines had reacted. The unreacted UPy-hexyl-COOH was removed via extraction with 2-isopropyl ether. And the access of NH_2-PNIPAM was removed using a methyl isocyanate bound to polystyrene resin and methyl-morpholine by stirring for 3 hours at RT. The solution was filtered and dried overnight, resulting in a white solid with 97.2 % yield. ^{1}H NMR (400 MHz, Chloroform-*d*) δ = 13.12 (s, 1H), 11.82 (s, 1H), 10.07 (s, 1H), 8.73 (s, 1H), 8.26 (s, 1H), 7.03 – 5.89 (m, 72H), 5.84 (s, 1H), 5.25 – 4.50 (m, 1H), 4.20 (t, *J* = 4.6 Hz, 2H), 4.03 (s, 17H), 3.64 (m, *J* = 1.5 Hz, 46H), 3.43 (m, *J* = 13.6, 7.5 Hz, 1H), 3.29 – 2.38 (m, 62H), 2.20 (m, *J* = 35.1 Hz, 12H), 1.44 – 0.93 (m, 147H). GPC: M_w 8888 g/mol, M_n 2339 g/mol, Đ = 3.8.

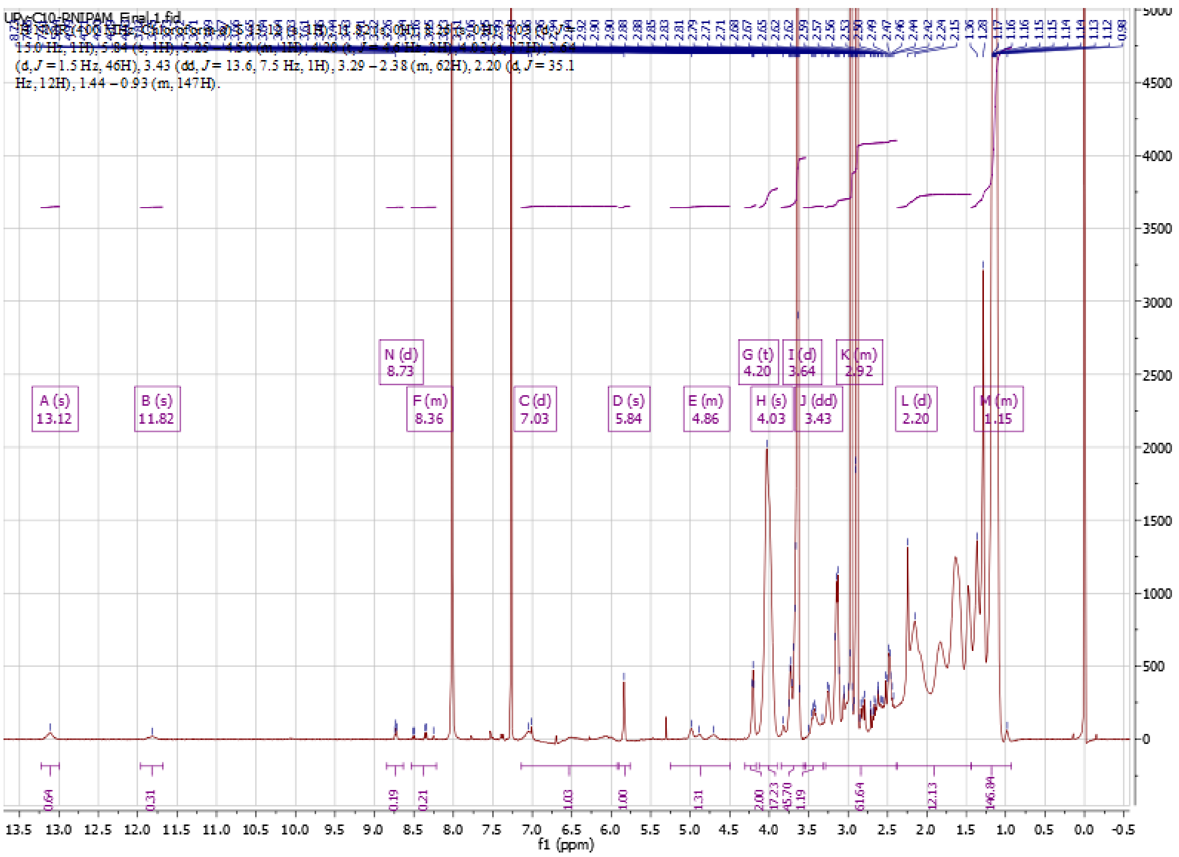

Synthesis of ***UPy-C₁₂-PNIPAM (4a)***: UPy-C_{12}-carboxylic acid (**20**) (3.3 mmol, eq) and 1.2 eq. NH_2-PNIPAM (Mn 2500 g/mol) were dissolved in dimethylformamide (DMF) and chloroform ($CHCl_3$) and the mixture was activated using 1.2 eq HATU, 3 eq. methyl-morpholine and stirred at 60 °C overnight. Ninhydrin staining was used to confirm that the primary amines had reacted. The unreacted UPy-hexyl-COOH was removed via extraction with 2-isopropyl ether. And the access of NH_2-PNIPAM was removed using a methyl isocyanate bound to polystyrene resin and methyl-morpholine by stirring for 3 hours at RT. The solution was filtered and dried overnight, resulting in a white solid in 97 % yield. (1.15 g, 96.8% yield). 1H NMR (400 MHz, Chloroform-d) δ = 13.09 (s, 1H), 11.75 (s, 1H), 10.04 (s, 1H), 8.59 (s, 1H), 8.25 (s, 1H), 7.22 – 5.91 (m, 22H), 5.83 (s, J = 4.6 Hz, 1H), 5.25 – 4.60 (m, 2H), 4.20 (t, 2H), 3.99 (s, 22H), 3.86 (t, J = 4.8 Hz, 11H), 3.64 (d, J = 4.3 Hz, 39H), 3.42 (s, 2H), 3.31 – 3.07 (m, 7H), 2.71 (d, J = 95.9 Hz, 21H), 2.38 – 1.53 (m, 38H), 1.49 – 0.71 (m, 129H). GPC: M_w 12479 g/mol, M_n 3897 g/mol, Đ = 3.2.

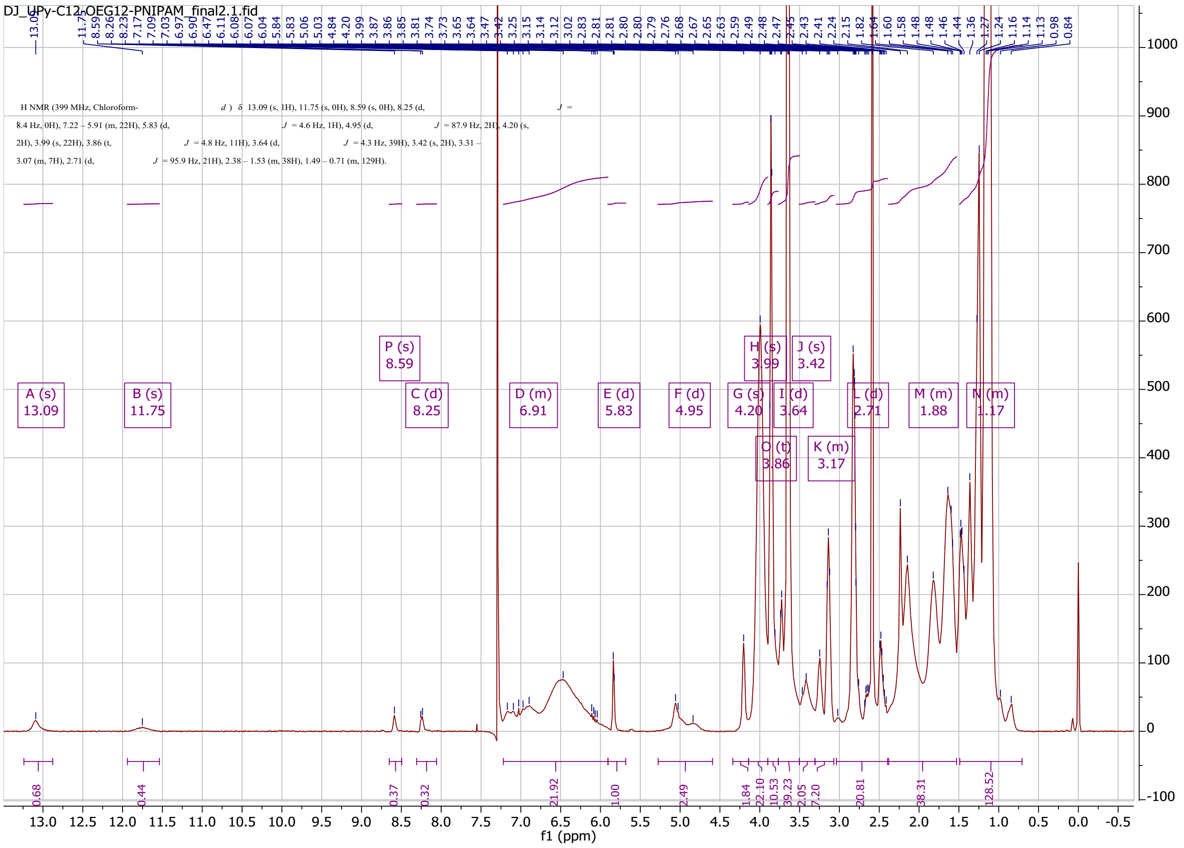

Synthesis of ***UPy-C$_{12}$-PNIPAM$_{5500}$ (4b)***: UPy-C$_{12}$-carboxylic acid (3.3 mmol, eq) and 1.2 eq. NH_2-PNIPAM (Mn 5500 g/mol) were dissolved in dimethylformamide (DMF) and chloroform ($CHCl_3$) and the mixture was activated using 1.2 eq HATU, 3 eq. methyl-morpholine and stirred at 60 °C overnight. Ninhydrin staining was used to confirm that the primary amines had reacted. The unreacted UPy-hexyl-COOH was removed via extraction with 2-isopropyl ether. And the access of NH_2-PNIPAM was removed using a methyl isocyanate bound to polystyrene resin and methyl-morpholine by stirring for 3 hours at RT. The solution was filtered and dried overnight, resulting in a white solid with 45.8 % yield (1.15 g, 98% yield). ^{1}H NMR (400 MHz, Chloroform-*d*) δ = 13.13 (s, 1H), 11.83 (s, 1H), 10.07 (s, 1H), 8.02 (s, 5H), 7.21 – 5.89 (m, 72H), 5.84 (s, 1H), 5.10 – 4.49 (m, 1H), 4.21 (t, J = 4.7 Hz, 2H), 4.00 (s, 54H), 3.65 (s, 47H), 3.43 (d, J = 6.4 Hz, 0H), 3.33 – 3.08 (m, 6H), 2.98 – 2.42 (m, 77H), 2.39 – 1.33 (m, 136H), 1.33 – 0.78 (m, 323H). GPC: M_w 28568 g/mol, M_n 7518 g/mol, Đ = 3.8.

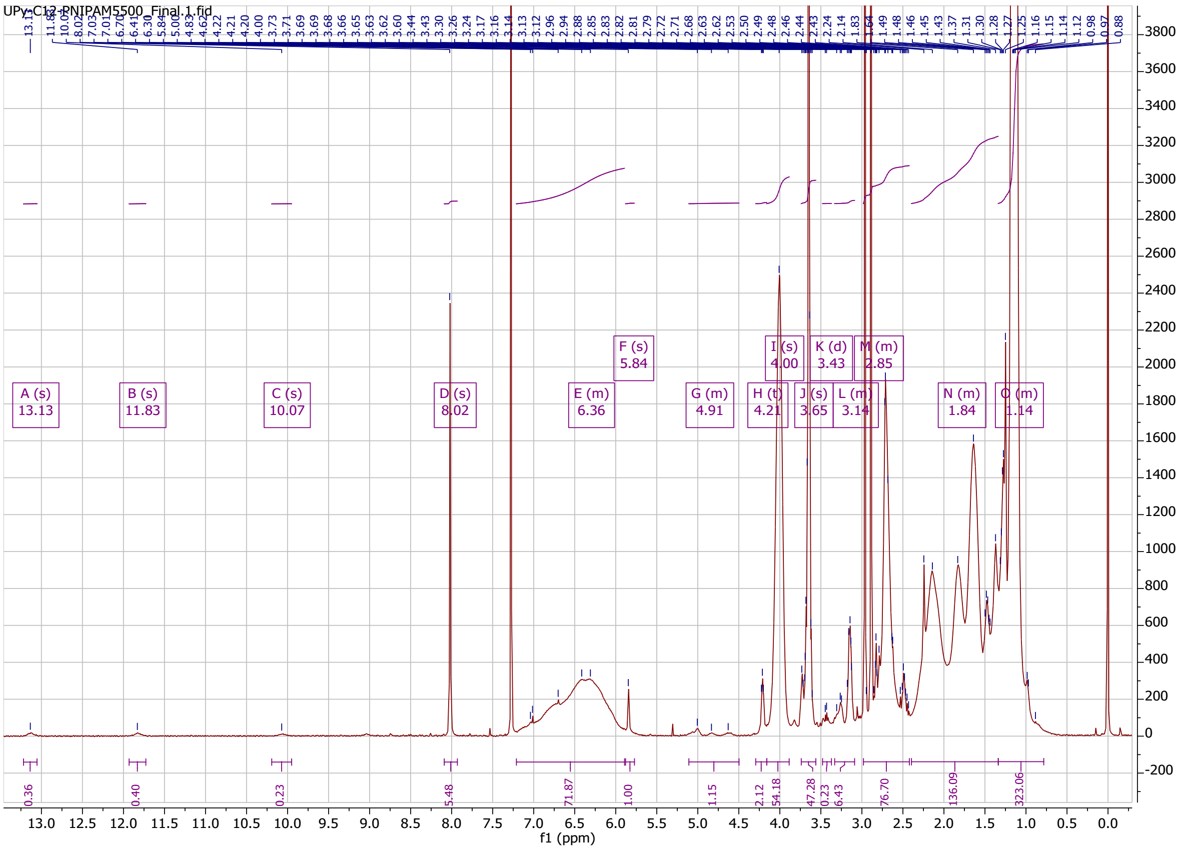

Synthesis of UPy-RGD-Cy5.

Synthesis of RGD peptide. The peptide pGGRGDSC was manually prepared using Solid Phase Peptide Synthesis (SPPS) and Fmoc chemistry. Of the Rink amide MBHA resin, 400 µmol was allowed to swell in NMP for 1 hour. Subsequently, the resin was washed with NMP (6x) and deprotected twice with a 20% piperidine solution in NMP for 5 minutes. The impurities were washed away with NMP (6x) and a cocktail of the desired amino acid was prepared of 200 mM amino acid, 1600 mM DIPEA, and 0.4 M HBTU in NMP (2:1:1). The cocktail was added to the resin and coupled for 30 min at room temperature. This cycle of washing-deprotection-washing-coupling was repeated for every amino acid. After the last amino acid was coupled, the fmoc protecting group was removed with an additional deprotection step, and the resin was washed with NMP and DCM, and dried. The resin was allowed to swell for 1 hour in DCM prior to cleavage. Cleavage reaction of the peptide was performed in TFA/H_2O/TIS/EDT (94:2:2:2) for 2.5 hours. Subsequently, the peptide was concentrated with a N_2 flow, precipitated in cold 20% Hexane/diethyl ether, incubated for 15 minutes at -20 °C, and centrifuged for 10 minutes at 2k RPM. The supernatant was removed, the pellet was redissolved in 10% ACN/H_2O and lyophilized. LCMS showed the desired peptide mass. LC-MS: pGRGDSC; Mw_{calc} = 687.73 g/mol, m/z_{obs} = 688.4 $[M+H]^+$ and 344.7 $[M+2H]^{2+}$.

Synthesis of RGD-Cy5. PBS, MQ water and DMF were degassed with argon. Sulfo-Cy5 maleimide (56.3 µmol, 1.7 eq) was dissolved in water/DMF and added to RGD peptide (32.2 µmol, 1 eq) dissolved in PBS. The reaction mixture was protected from light and agitated overnight at room temperature. The reaction mixture was purified using gel filtration with Sephadex G10 yielding RGD-Cy5 (29 mg, 80% yield). LCMS: Mw_{calc} = 1451.63 g/mol, m/z_{obs} = 1453.5 $[M+H]^+$ and 727.2 $[M+2H]^{2+}$.

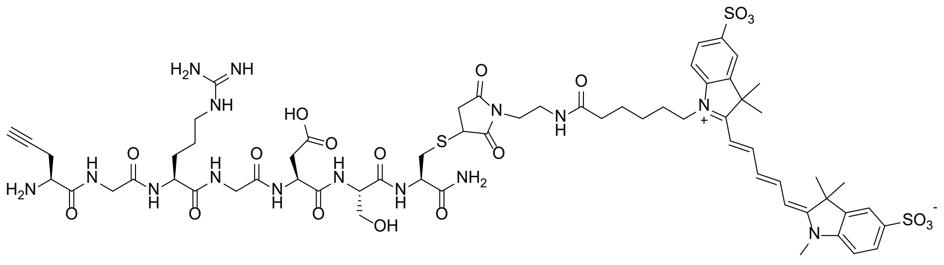

Synthesis of UPy-OEG$_{11+4}$-N$_3$. UPy-OEG$_{11}$-NH$_2$ was synthesized as previously reported.[1] UPy-OEG$_{11}$-NH$_2$ (107 µmol, 1 eq) was dissolved in dry DCM, to which triethylamine (160 µmol, 1.5 eq) was added. Subsequently, NHS-OEG$_4$-N$_3$ (107 µmol, 1 eq) dissolved in DCM was added. The reaction mixture was stirred overnight at room temperature, and regularly checked with LCMS. After full conversion, the reaction mixture was precipitated in cold 20% hexane/diethyl ether and centrifuged. The pellet was dissolved in 20% ACN/H_2O and lyophilized yielding UPy-OEG$_{11+4}$-N$_3$ with minor impurities in 102% yield. LCMS: Mw_{calc} = 1338.65 g/mol, m/z_{obs} = 1338.8 $[M+H]^+$ and 670.2 $[M+2H]^{2+}$.

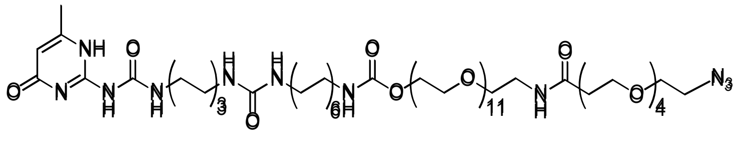

Synthesis of ***UPy-GRGDS-Cy5***. UPy-OEG$_{11+4}$-N$_3$ (6.9 µmol, 1 eq) was dissolved in DMF and added to RGD-Cy5 (7.5 µmol, 1 eq) dissolved in MQ water. Separately, a reaction cocktail containing copper sulfate (5 mM), aminoguanidine (20 mM), Bim(py)$_2$ (3mM) and fresh sodium ascorbate (20 mM) was prepared and added to the UPy-RGD mixture. The reaction mixture was stirred overnight at room temperature, and purified on a reversed phase C18 column using a gradient of 5-100% acetonitrile in water yielding **UPy-RGD-Cy5** in 46% yield with minor impurities. LCMS: Mw_{calc} = 2790.28 g/mol, m/z_{obs} = 1396.1 $[M+2H]^{2+}$, 931.1 $[M+3H]^{3+}$ and 698.6 $[M+4H]^{4+}$.

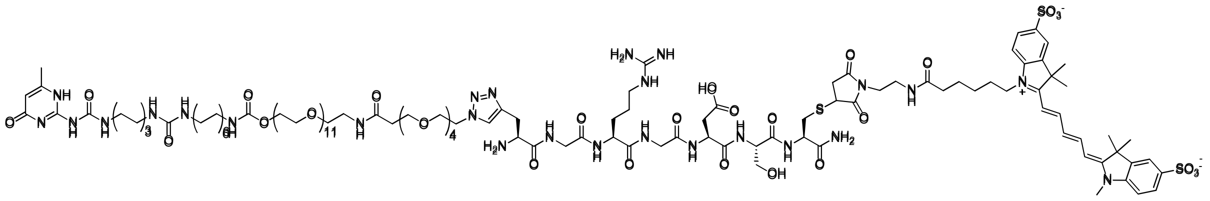

4. Cell culture

Human-induced pluripotent stem cells (hiPSC) were cultured in Essential 8 medium (Thermofisher Scientific) on a rh-VTN (Thermofisher Scientific) coating. The medium for hiPSCs was refreshed daily. Cells were cultured at 37 °C and 5% CO_2. hiPSCs were passaged every 3-4 days. For hiPSCs, the Essential 8 medium was supplemented with RevitaCell™ Supplement during the initial 24 hours after passaging. hiPSCs were incubated with CellTracker™ Green CMFDA Dye (Thermofisher Scientific) at a concentration of 10 µM for 30 minutes prior to enzymatic dissociation using TrypLE™ Select Enzyme (Thermofisher Scientific).

4.1. Microfluidic device production

Polydimethylsiloxaan (PDMS) devices for microfluidic hydrogel droplet production were fabricated by mixing SYLGARD® 184 PDMS with SYLGARD® 184 curing agent (Merck) at 10:1. The mixture was poured onto a silicon wafer containing the device layout (**Figure S1**) and allowed to cure for two hours at 65 °C. After curing the PDMS was removed and 1 mm holes were punched for the inlets and outlet. The PDMS was bonded channels-down to glass slides via plasma ashing (Emitech, K1050X). After bonding the channels were treated with 5% perfluorooctyltriethoxysilane in HFE-7500 (Fluorochem), incubated for one hour at 65 °C, flushed with HFE-7500, and incubated overnight at 65 °C for thermal bonding.

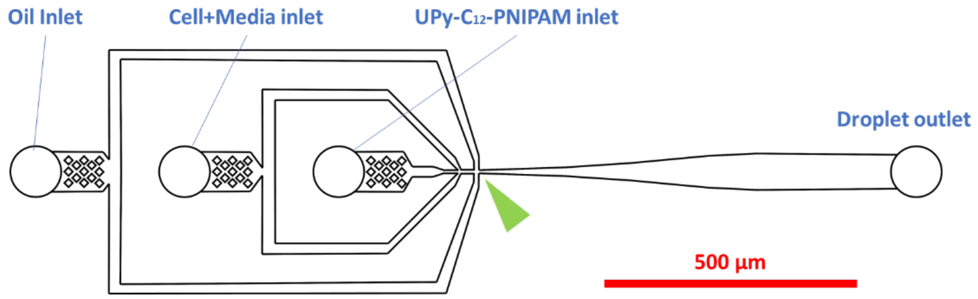

Figure S1. Layout of microfluidic device. Green arrow represents channel intersection where droplet formation occurs.

4.2. Cells encapsulation in hydrogel droplets

hiPSCs were resuspended in their respective culture medium at a concentration of $4.0 \cdot 10^6$ cells/mL. Furthermore, the hiPSC suspension was supplemented with RevitaCell™

Supplement. Microfluidic droplets containing **UPy-C12-PNIPAM** and **UPy-GRGDS-Cy5** were generated using a tip-loading approach, meaning that the cell solution and hydrogel solution were drawn into a 200 µl pipet tip and loaded to the inlets in the PDMS chip as has been described.[3] A neMESYS pump (Cetoni) and tubing filled with mineral oil (Merck) was used as hydraulic system. 2.5% PicoSurf (SphereFluidics) in HFE 7500 (Fluorochem) was used to form droplets and flushed into the oil inlet, while cells and the hydrogel were flushed from the inner two inlets. Flow speed used was 30 µl/min for oil and 5 µl/min for cells and hydrogel. The cell concentration at the droplet formation point was $2.0 \cdot 10^6$ cells/mL. A 139 mM **UPy-C12-PNIPAM** and 2.7 mM **UPy-GRGDS-Cy5** polymer solution was loaded with 1.25 wt% as end concentration. The droplets were collected in an Eppendorf tube from the outlet, and 150 µl of PBS was added on top of the emulsions to prevent evaporation of HFE oil. After encapsulation, the cells were incubated at 37 °C. Imaging was performed at time point 3 and 24 hours using a Leica TCS SP8X confocal microscope (Leica Biosystems) at RT and 37 °C.

5. Additional results

5.1. Cryogenic Transmission Electron Microscopy (cryo-TEM)

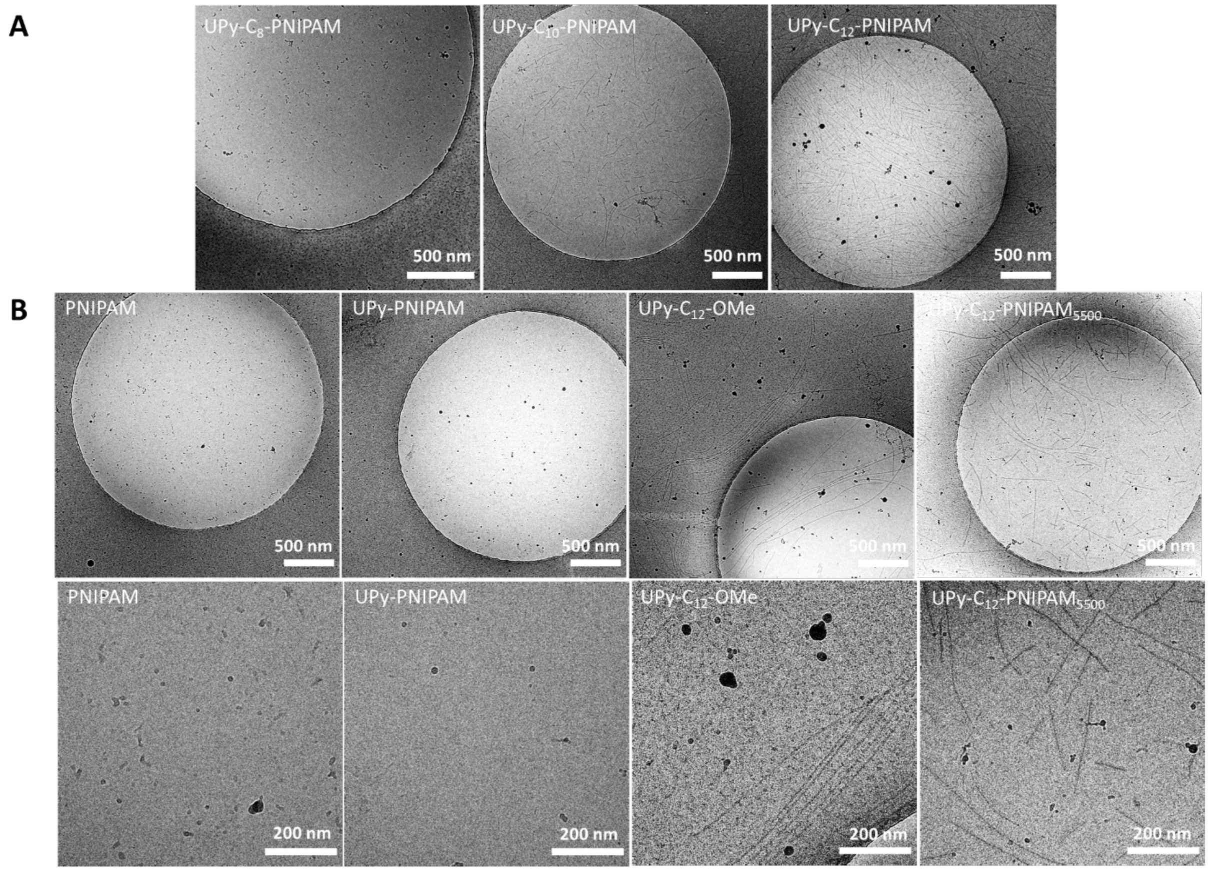

Figure S2. Structural characterization of the supramolecular PNIPAM polymers in solution (50 µM). A. *Cryo-TEM images (Zoom out) of globular structures in* ***UPy-C$_8$-PNIPAM****, globular and fibrillar structures in* ***UPy-C$_{10}$-PNIPAM*** *and fibrillar structures for* ***UPy-C$_{12}$-PNIPAM*** *were observed.* ***B.*** *PNIPAM and* ***UPy-PNIPAM*** *demonstrate no assembly or pre-structural organization due to the hydrophilic nature of the PNIPAM. Micrometer long fibers were displayed for* ***UPy-C$_{12}$-OMe****. For* ***UPy-C$_{12}$-PNIPAM$_{5500}$*** *fibrillar structures were observed.*

5.2. Total internal reflection fluorescence (TIRF)

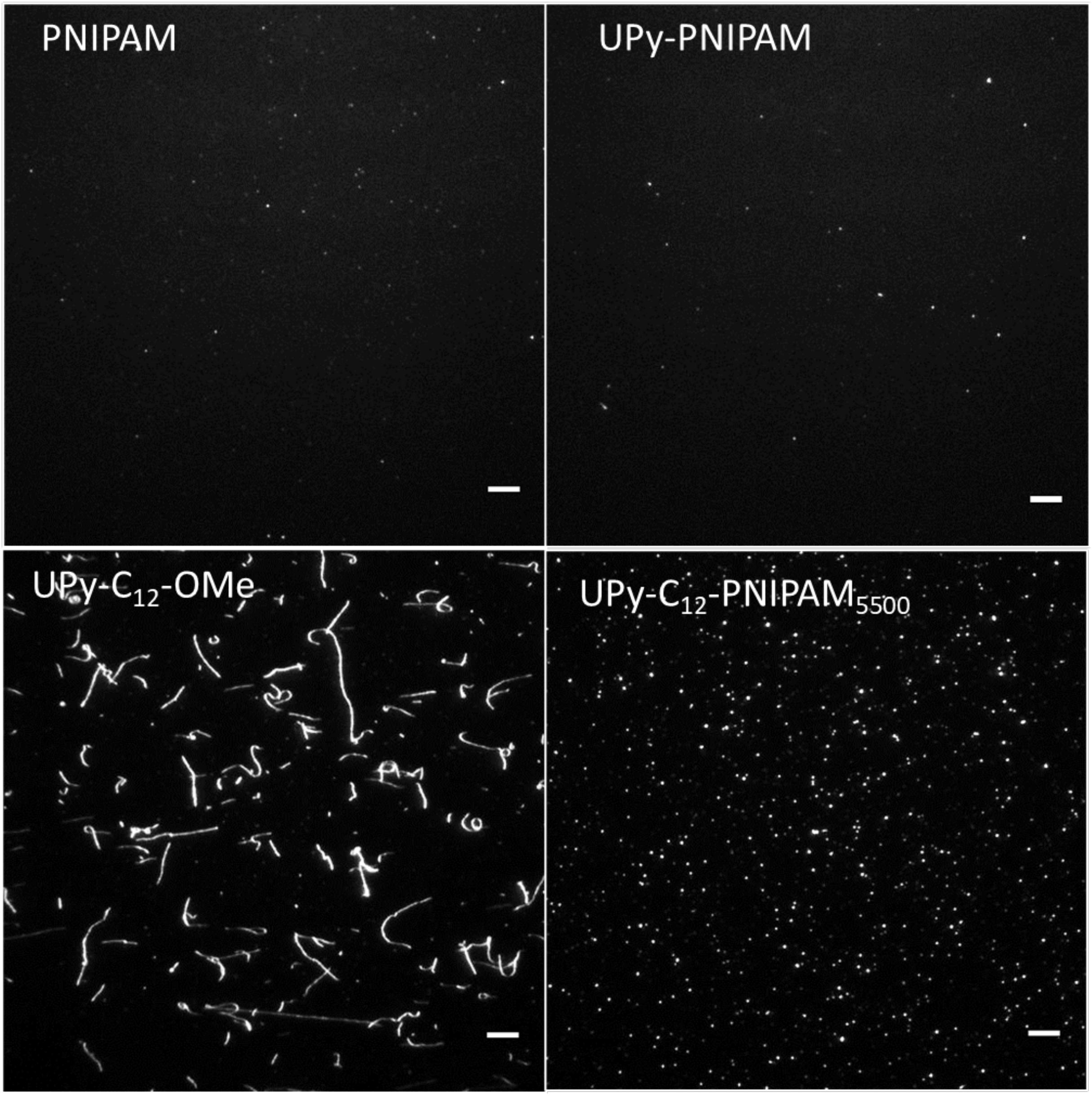

Figure S3. Structural characterization of UPy-C_n-PNIPAM in solutions using TIRF. *Very minor aggregates were observed for PNIPAM and **UPy-PNIPAM**. **UPy-C_{12}-OMe** consisted of micrometer long fibers. Small fiber-like structures were observed for the **UPy-C_{12}-PNIPAM$_{5500}$** polymers. The increase of the alkyl-spacers showed an increase of the fiber size which indicates dominating UPy-stacking. The scale bars represent 5 µm.*

5.3. Nile red fluorescence measurements

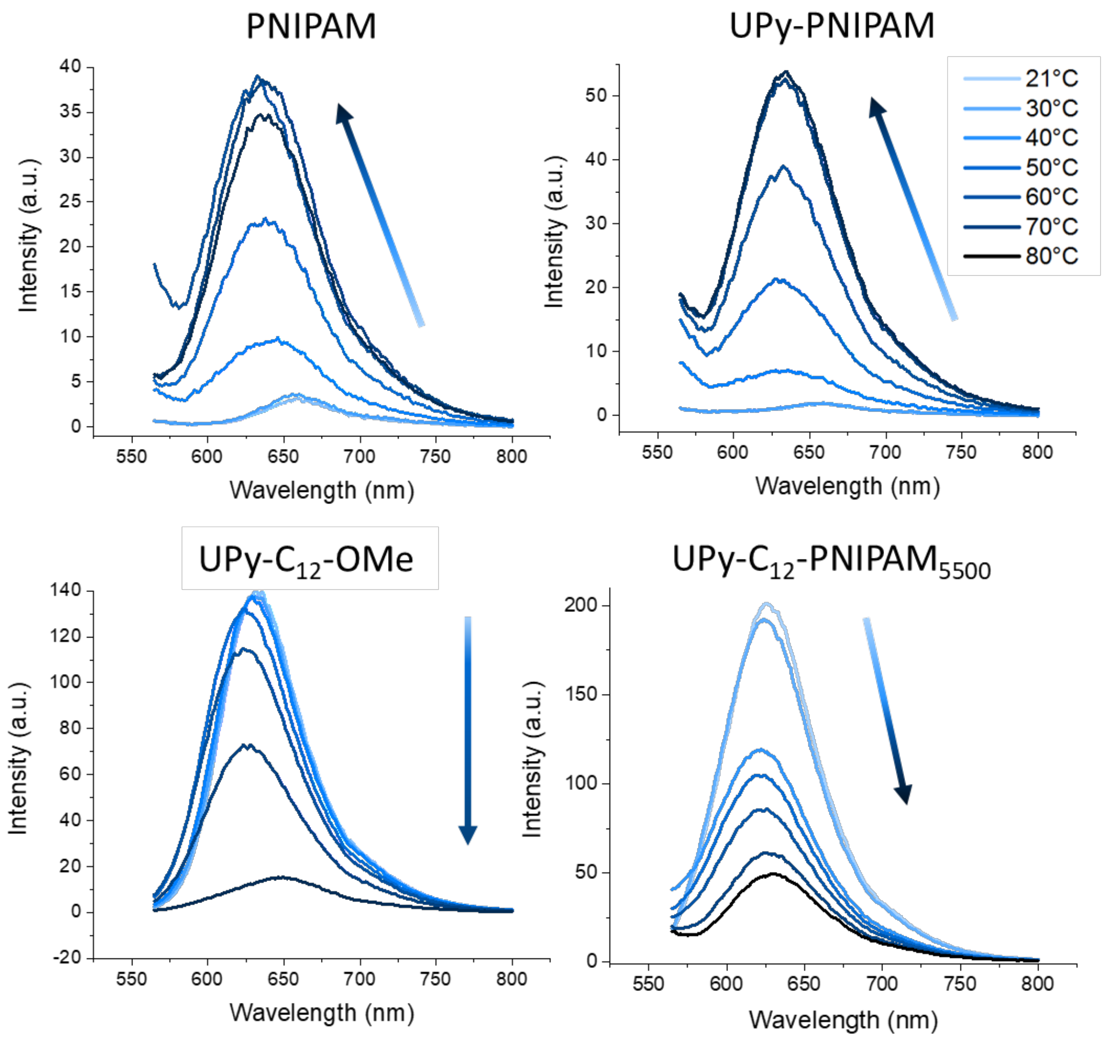

Figure S4. UPy-Cₙ-PNIPAM structural assembly in solution at different temperatures. *Supramolecular polymer formation was probed using a hydrophobic dye nile red (NR). PNIPAM demonstrates temperature responsive behavior, as the polymer becomes more hydrophobic which results in increase of NR intensity. A similar response is obtained for* ***UPy-PNIPAM****, without an alkyl spacer. In contrast,* ***UPy-C$_{12}$-PNIPAM$_{5500}$*** *and* ***UPy-C$_{12}$-OMe*** *showed a decrease in the fluorescent intensity upon temperature increase. Showing that upon the incorporation of a long alkyl-spacer UPy fibers can be formed, which are dominating over the PNIPAM response.*

5.4. Rheology

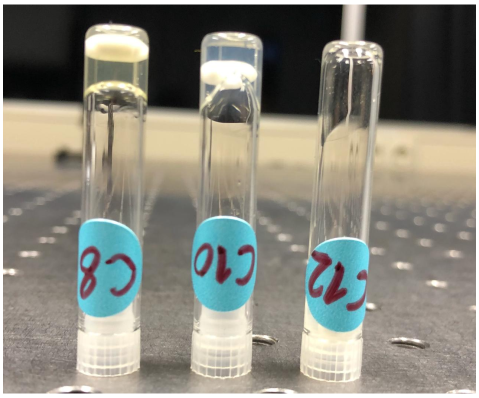

Figure S5. Digital photograph showing different 10 wt% UPy-C$_n$-PNIPAM compositions subjected to the inverted-vial test at room temperature. From left to right: **UPy-C$_8$-PNIPAM**, **UPy-C$_{10}$-PNIPAM** and **UPy-C$_{12}$-PNIPAM.**

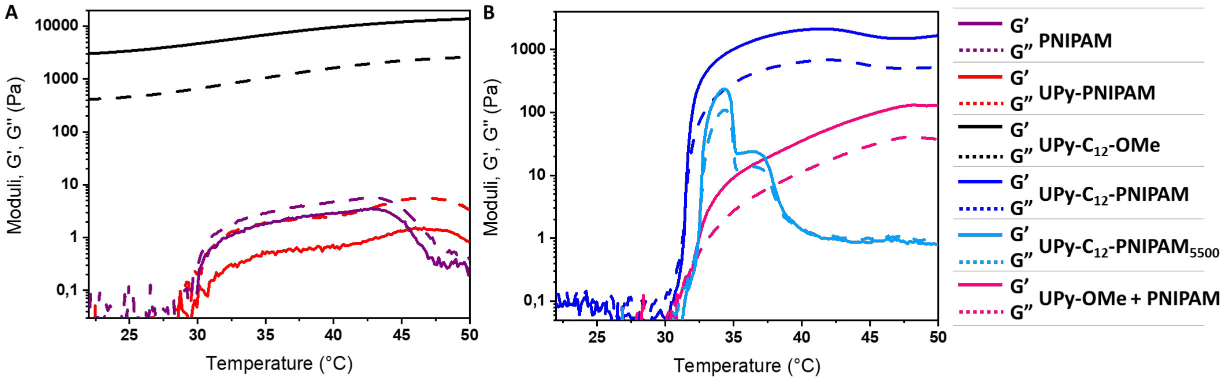

Figure S6. Thermoresponsive mechanical behavior of 10 wt% hydrogels. A. UPy-OMe *showed visco-elastic behavior with a small increase of moduli over time. PNIPAM and* ***UPy-PNIPAM*** *remained a liquid upon increase of temperature (G'' > G'). **B. UPy-C$_{12}$-PNIPAM$_{5500}$** became a soft material upon increase of temperature and ultimately liquifies (G'' > G'). The mixture of **UPy-C$_{12}$-OMe** and pristine PNIPAM exhibited a weaker thermo-responsiveness compared to UPy-C$_n$-PNIPAM polymers.*

5.5. Small angle X-ray scattering

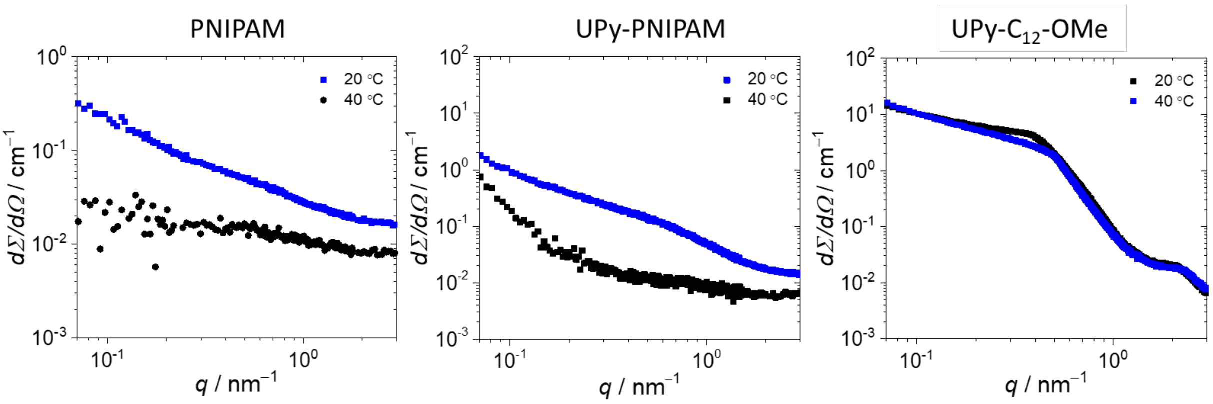

Figure S7. Representative small-angle X-ray scattering (SAXS) profiles of 10 wt% hydrogels in dependence of temperature. *Comparison of the scattering intensities of the 10 wt% PNIPAM.* ***UPy-PNIPAM*** *and* ***UPy-C*** $_{12}$ ***-OMe*** *to probe the structural organization and the mechanism of temperature transition.*

5.6 Rheology measurements varying concentration and assembly time

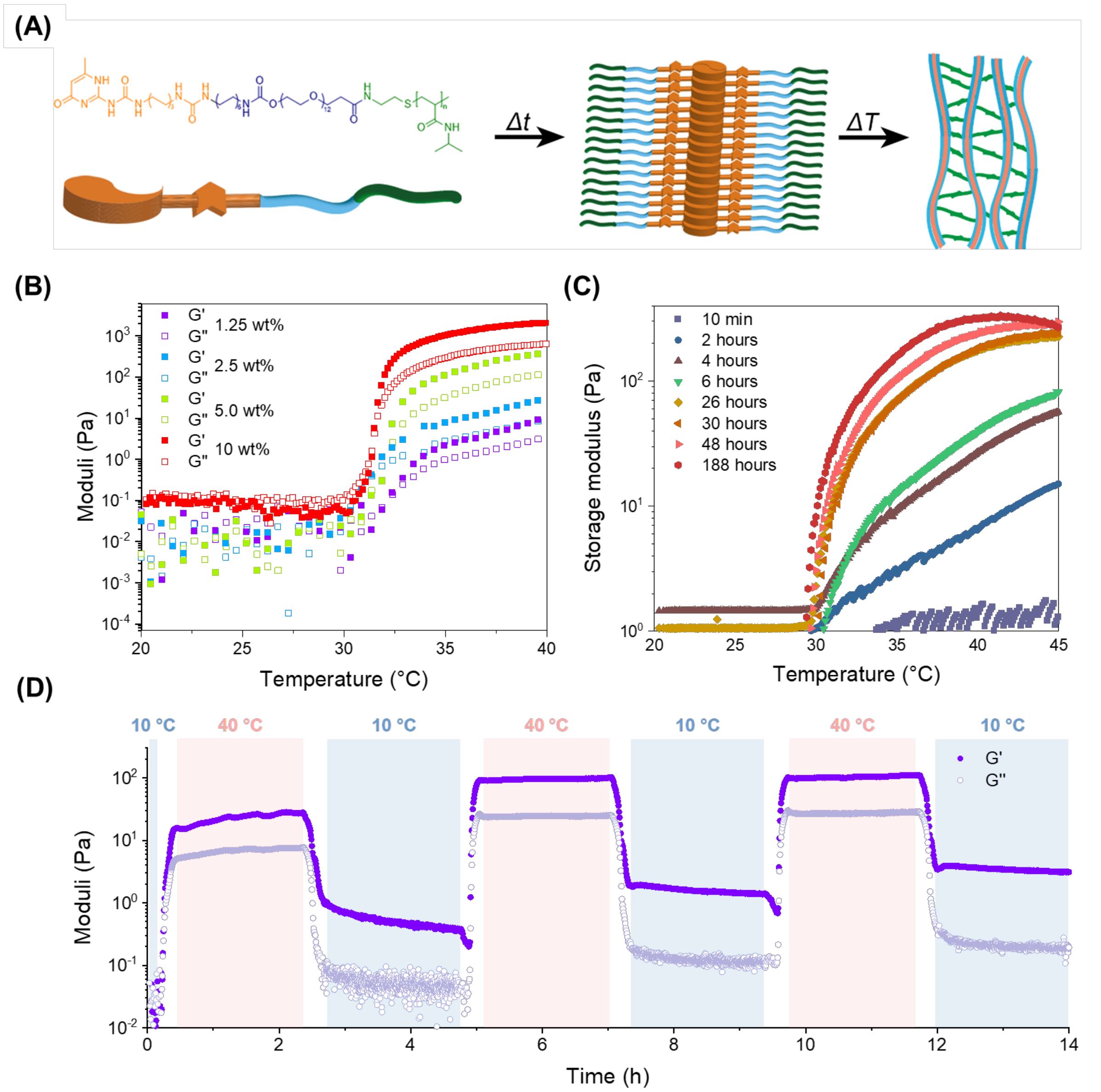

Figure S8. Rheological analysis of UPy-C$_{12}$-PNIPAM at different concentrations and assembly time. A. *Schematic overview of* ***UPy-C$_{12}$-PNIPAM*** *stacking into fibers. Mechanical interlocking occurs when the PNIPAM tail is heated beyond its cloud point.* ***B.*** *Rheological measurements demonstrated tunable stiffness in concentrations of* ***UPy-C$_{12}$-PNINPAM****. Higher stiffnesses can be achieved using higher concentrations of* ***UPy-C$_{12}$-PNIPAM****.* ***C.*** *The increase of pre-assembly time prior to rheological measurement increased the stiffness of the* ***UPy-C$_{12}$-PNIPAM*** *above the LCST due to ordering of the monomers into stacks (all samples 1.68 wt%).* ***D.*** *Reversible behavior was observed in* ***UPy-C$_{12}$-PNIPAM*** *(1.25 wt%) during multiple heating and cooling cycles. Samples were heated and cooled between 10 °C and 40 °C (with a ramp of 1 °C/min). Only a strong elastic gel is formed above the LCST.*

6. References

1. De Feijter, I. *et al.* Solid-Phase-Based Synthesis of Ureidopyrimidinone-Peptide Conjugates for Supramolecular Biomaterials. *Synlett* **26**, 2707–2713 (2015).

2. Wu, H. Correlations between the Rayleigh ratio and the wavelength for toluene and

benzene. *Chem. Phys.* **367**, 44–47 (2010).

3. Sinha, N., Subedi, N., Wimmers, F., Soennichsen, M. & Tel, J. A Pipette-Tip Based Method for Seeding Cells to Droplet Microfluidic Platforms. *J. Vis. Exp.* e57848 (2019). doi:10.3791/57848
